# *N* ^6^-Methyladenosine Methylomic Landscape of Lung Tissues in Murine Acute Allergic Asthma

**DOI:** 10.3389/fimmu.2021.740571

**Published:** 2021-10-19

**Authors:** Fangzhou Teng, Weifeng Tang, Tulake Wuniqiemu, Jingjing Qin, Yaolong Zhou, Xi Huang, Shiyuan Wang, Xueyi Zhu, Zhao Tang, La Yi, Ying Wei, Jingcheng Dong

**Affiliations:** ^1^ Department of Integrative Medicine, Huashan Hospital, Fudan University, Shanghai, China; ^2^ Institutes of Integrative Medicine, Fudan University, Shanghai, China

**Keywords:** asthma, mouse, lung tissue, OVA, m6A, *N* ^6^-methyladenosine, MeRIP-seq

## Abstract

Allergic asthma is well known as a common respiratory disorder comprising an allergic inflammatory nature and excessive immune characteristic. *N* ^6^-methyladenosine (m6A) methylation is an RNA epigenetic modification that post-transcriptionally regulates gene expression and function by affecting the RNA fate. Currently, m6A methylation is gaining attention as a mechanism of immunoregulation. However, whether m6A methylation engages the pathological process of asthma remains uncertain. Here, we present the m6A methylomic landscape in the lung tissues of ovalbumin-induced acute asthma mice using MeRIP-seq and RNA-seq. We identified 353 hypermethylated m6A peaks within 329 messenger RNAs (mRNAs) and 150 hypomethylated m6A peaks within 143 mRNAs in the lung tissues of asthmatic mice. These differentially methylated mRNAs were found to be involved in several immune function-relevant signaling pathways. In addition, we predicted 25 RNA-binding proteins that recognize the differentially methylated peak sites by exploring public databases, and the roles of these proteins are mostly related to mRNA biogenesis and metabolism. To further investigate the expression levels of the differentially methylated genes, we performed combined analysis of the m6A methylome and transcriptome data and identified 127 hypermethylated mRNAs (107 high and 20 low expression) and 43 hypomethylated mRNAs with differential expressions (9 high and 34 low expression). Of these, there are a list of mRNAs involved in immune function and regulation. The present results highlight the essential role of m6A methylation in the pathogenesis of asthma.

## Introduction

*N* ^6^-methyladenosine (m6A) methylation is an RNA epigenetic modification that is one of the most abundant modifications in eukaryotic messenger RNA (mRNA) (1). Several scholars have argued that m6A modification has a critical role in regulating mRNA expression (2), stability (3), and translation (4). These regulations affect the diverse vital activities of living systems. Numerous research studies have confirmed the importance of m6A-modified mRNAs in multiple physiological functions and disease pathologies throughout life, including, but not limited to, regulation of the formation of various tissues such as cerebral cortex development (5), ocular angiogenesis (6), and adipogenesis (7) and the regulation of cell differentiation and function such as in hematopoietic stem cells (8), pancreatic β cells (9), skeletal muscle cells (10), and spermatozoa (11). Besides, changes in the m6A methylation levels of mRNAs also contribute to a variety of tumors, such as gastric (12), breast (13), lung (14), thyroid (15), and bladder (16) cancers, and non-tumor diseases, such as heart failure (17), type 2 diabetes (18), depressive disorder (19), and kidney injury (20). Recently, studies have confirmed that m6A RNA modification is closely related to the regulation of immune function (21). It has been found that m6A modification can affect the differentiation of macrophages (22), the homeostasis and function of T cells (23), and the activation of dendritic cells by regulating the stability of mRNAs. In addition, m6A modification is also concerned with various inflammatory responses in diseases such as spontaneous colitis (24), alcoholic kidney injury (25), and coronary artery disease (26).

The pathogenesis of asthma is predominantly attributed to the excessive immune response of T helper cell type 2 (Th2) and the production of substantial amounts of inflammatory cytokines [interleukin 4 (IL-4), IL-5, and IL-13] (27, 28). These cytokines mediate eosinophilic infiltration, airway hyperresponsiveness, and mucus and immunoglobulin E (IgE) overproduction (27, 29). Ovalbumin (OVA) extracted from chicken eggs is a well-known allergen and was applied to induce robust allergic lung inflammation in mice (30). Since OVA can effectively induce Th2 immune response, airway eosinophil infiltration, and airway hyperreactivity in mice (31), OVA induction has become a classic method of asthma modeling. To date, the role of other epigenetic modifications (DNA methylation, histone modification, and miRNA) in asthma has been well identified (32–34). Previous studies have also shown that m6A modification has an essential role in the regulation of immune function and inflammatory response; however, the relationship between m6A methylation and asthma remains to be elucidated.

For further insight into the role of m6A in asthma, we determined the distribution patterns of transcriptome-wide methylated sites and identified the differentially methylated mRNA transcripts in the lung tissues following OVA-induced asthma *via* methylated RNA immunoprecipitation sequencing (MeRIP-seq) and RNA sequencing (RNA-seq). To explore the potential mechanisms by which differentially methylated mRNAs might function, we next predicted the RNA-binding protein (RBP) candidates that might bind to the differential methylation site. Lastly, we analyzed the gene expressions of the differentially methylated mRNAs by combining the MeRIP-seq and RNA-seq data in order to explore whether the methylation levels of mRNAs affect their corresponding gene expression levels. Our study uncovers the underlying mechanism of m6A methylation in asthma.

## Results

### Mouse Model of OVA-Induced Allergic Asthma

**Figure 1:**
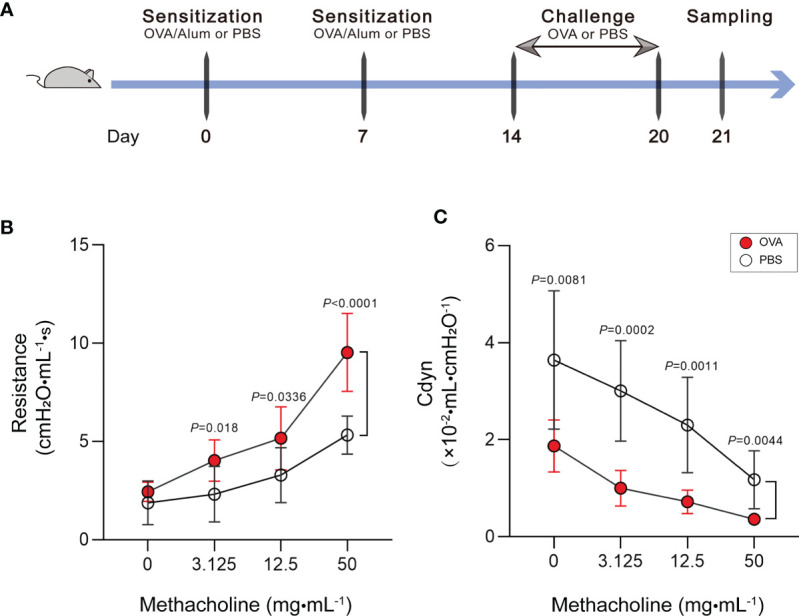
Increase of airway hyperresponsiveness in OVA-induced asthma. **(A)** Schematic diagram of the establishment of acute allergic asthma mouse model. **(B, C)** Lung resistance **(B)** and dynamic compliance **(C)** (*C*_dyn_) in response to increasing doses of methacholine. Values are presented as the means ± SD (*n* = 11, two-way mixed-design ANOVA). *OVA*, ovalbumin; *Alum*, aluminum hydroxide; *PBS*, phosphate-buffered saline.

Our study is based on the classic OVA model of allergic asthma. The schematic design of the animal modeling is shown in **Figure 1A**. Airway hyperresponsiveness is the most typical pathological feature of allergic asthma. To explore the ability of OVA to effectively induce airway hyperresponsiveness in mice, we tested the pulmonary function of mice. In the OVA-induced asthma mouse model (hereafter abbreviated as OVA group), lung resistance was increased (**Figure 1B**) and the dynamic compliance (*C*_dyn_) (**Figure 1C**) was significantly decreased at 3.125, 12.5, and 50 mg/ml methacholine compared with those of phosphate-buffered saline (PBS)-treated control mice (hereafter abbreviated as PBS group).

**Figure 2:**
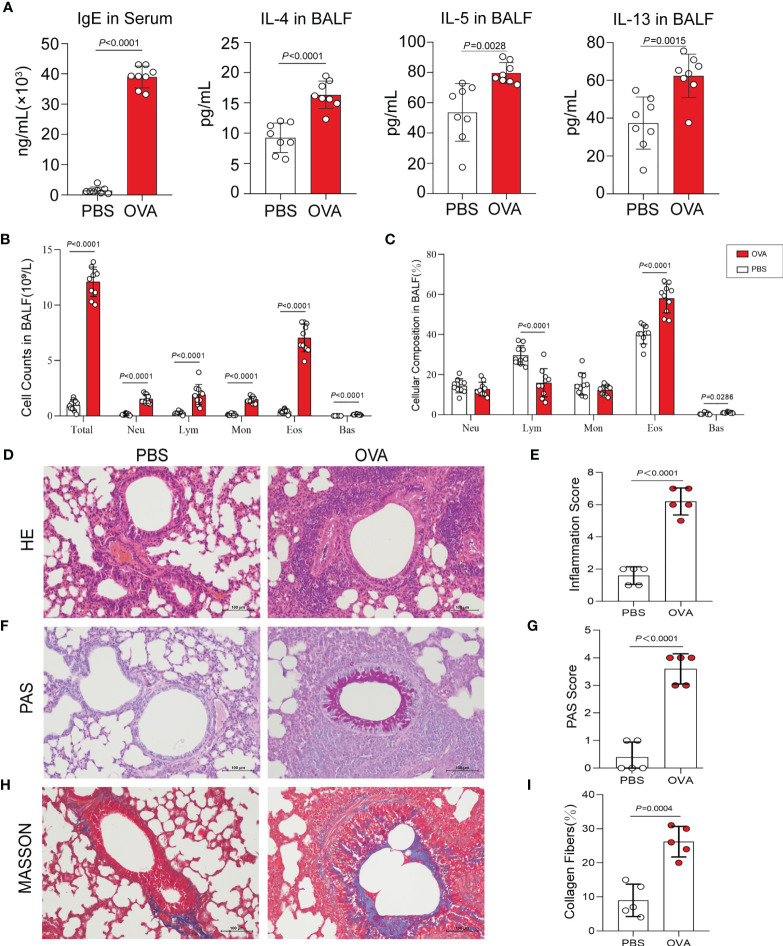
Allergic inflammation of airway induced by ovalbumin (OVA). **(A)** Levels of IgE in serum and IL-4, IL-5, and IL-13 in bronchoalveolar lavage fluid (BALF) (*n* = 8). **(B, C)** Cell count **(B)** and percentage **(C)** of leukocytes in BALF (*n* = 10). **(D)** Representative images of hematoxylin and eosin (HE) stain on lung sections. **(E)** Inflammatory cell infiltration was quantified by the inflammation score. **(F)** Representative images of periodic acid–Schiff (PAS) stain. **(G)** Mucus secretion was quantified by PAS scores. **(H)** Representative images of Masson’s trichrome (MT) stain. **(I)** Collagen deposition was quantified by the percentage of collagen fibers area (*n* = 5). *Scale bar*, 100 μm. Values are presented as the means ± SD (unpaired and two-tailed Student’s *t*-test). *Total*, total leukocytes; *Neu*, neutrophils; *Lym*, lymphocytes; *Mon*, monocytes; *Eos*, eosinophils; *Bas*, basophils.

Th2-type immune response is crucial for triggering the development of airway hyperresponsiveness. Therefore, we next examined airway inflammation in the OVA group using ELISA, hematoxylin and eosin (HE) staining, and leukocyte classification and counting. In ELISA (**Figure 2A**), the levels of IgE in serum and IL-4, IL-5, and IL-13 in bronchoalveolar lavage fluid (BALF) were measured in mice in the OVA group at significantly higher concentrations than that in PBS control mice. Meanwhile, we classified and counted the leukocytes in BALF, and the results indicated distinctly increased numbers of total leukocytes (Total), neutrophils (Neu), lymphocytes (Lym), monocytes (Mon), eosinophils (Eos), and basophils (Bas) (**Figure 2B**) and rising percentages of Lym, Eos, and Bas (**Figure 2C**) in the OVA group compared with those in the PBS group. Furthermore, HE staining of lung tissue sections showed an increased inflammatory cell infiltration around the airway and blood vessels in OVA-induced mice (**Figure 2D**), and the corresponding histological inflammation score (**Figure 2E**) was higher in the OVA group compared with that of the PBS group.

Additionally, we performed periodic acid–Schiff (PAS) and Masson’s trichrome (MT) staining on the lung sections. PAS staining and positive scores revealed increased airway mucus secretion in the OVA group, which was implicated in goblet cell hyperplasia (**Figures 2F, G** **)**, and the results of MT staining showed that the increase in collagen fiber deposition in the airways of mice in the OVA group confirmed the change of the airway structure and the occurrence of airway remodeling (**Figures 2H, I** **)**.

### Overview of Sequencing Data

After six MeRIP-seq libraries and six RNA-seq libraries were sequenced, an average of 92,112,289 raw reads (13.82 GB data) from MeRIP-seq and 79,562,999 raw reads (11.93 GB data) from RNA-seq were obtained (**Supplementary Table S1**). Then, the reads that contained adaptor contamination, low-quality bases, and undetermined bases were filtered out and clean reads were obtained. Specifically, we collected an average of 89,544,192 valid MeRIP-seq reads (12.31 GB data) and 77,517,266 valid RNA-seq reads (10.58 GB data) (**Supplementary Table S1**). Further analysis found that an average of 77,085,259 (93.05%) clean reads were mapped to the mouse reference genome. Of these, an average of 11,444,146 (13.84%) were multi-mapped reads and 65,641,113 (79.21%) were unique mapped reads (**Supplementary Table S2**). Having analyzed the mapped reads in different genomic regions, we found approximately 58.41% of reads mapped to exons, 39.8% mapped to introns, and 1.79% mapped to intergenic regions (**Supplementary Table S3**).

### Distribution Characteristics of m6A Methylated Peaks in Asthma

**Figure 3:**
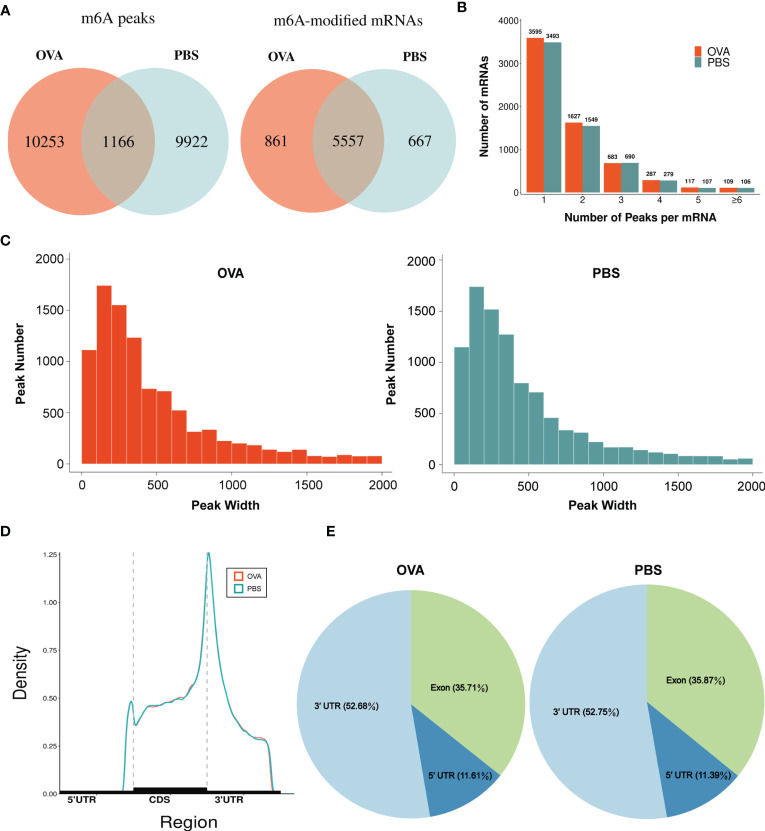
Characteristics of *N* ^6^-methyladenosine (m6A) methylation in lung tissues of the phosphate-buffered saline (PBS) and ovalbumin (OVA) groups. **(A)** Number of group-specific and shared m6A peaks and m6A-modified mRNAs. **(B)** Number of mRNAs containing different numbers of m6A peaks. **(C)** Number of peaks with different widths. **(D)** Distribution density of m6A peaks in the 5' UTR, CDS, and 3' UTR regions. **(E)** Percentage of m6A peaks in the 5' UTR, exon, and 3' UTR regions.

By comparing the distributions of the MeRIP-seq and RNA-seq reads in the lung tissues of mice in the OVA and PBS groups, we identified 27,292 m6A peaks within 14,118 transcripts in the OVA group (**Supplementary Table S4**) and 26,102 m6A peaks within 13,564 transcripts in the PBS group (**Supplementary Table S5**). We next analyzed the enrichment of the m6A peaks in the mRNA transcripts and found that 11,419 m6A peaks were enriched in 6,418 mRNA transcripts of the OVA group (**Supplementary Table S6**) and 11,088 m6A peaks were enriched in 6,224 mRNA transcripts of the PBS group (**Supplementary Table S7**). Further analysis revealed an overlap of 1,166 peaks and 5,557 m6A-modified mRNAs between the two groups. The OVA group had 10,253 unique m6A peaks and 861 unique m6A-modified mRNAs, while the PBS group had 9,922 unique m6A peaks and 667 unique m6A-modified mRNAs (**Figure 3A**). Meanwhile, the distribution characteristics of the peak frequency and width within the mRNA transcripts were analyzed in the two groups. The results showed that the majority of m6A-modified mRNAs (3,595 in the OVA group and 3,493 in the PBS group) contained only one peak site (**Figure 3B**), and the widths of the majority of peak sites (7,088 in the OVA group and 7,188 in the PBS group) were within 600 bp (**Figure 3C**).

**Figure 4:**
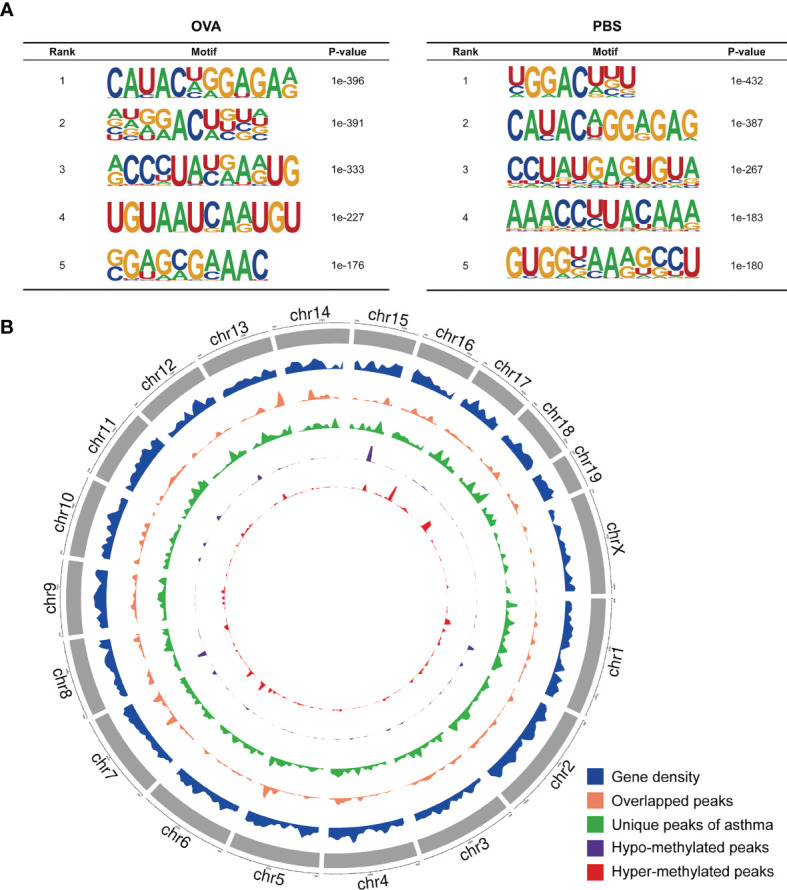
Landscape of *N* ^6^-methyladenosine (m6A)-modified transcripts in ovalbumin (OVA)-induced asthma. **(A)** Top five sequence motifs in the lung tissues from the OVA and phosphate-buffered saline (PBS) groups. **(B)** Density distribution of the m6A peaks along the chromosome. The first track (*blue circle*) indicates the gene density in the lung tissues of the two groups. The second track (*orange circle*) indicates the frequency distribution of overlapping m6A peaks between the OVA and PBS groups. The third track (*green circle*) indicates the frequency distribution of unique m6A peaks in the OVA group. The fourth (*purple circle*) and fifth (r*ed circle*) tracks indicate the frequency distribution of the hypo- and hypermethylated peaks, respectively. *chr*, chromosome.

We also investigated the genic region distributions of all m6A peak locations in asthmatic and normal lung tissues. **Figure 3D** shows that the peaks in both the OVA and PBS groups were dominantly distributed in the coding sequence (CDS) near the stop codon. Furthermore, the distribution patterns of the m6A peaks in genic regions of the two groups also showed similar trends. The peaks were abundant in the 3' untranslated (UTR) region (52.68% in the OVA group and 52.75% in the PBS group) and the exon region (35.71% and 35.87%, respectively), followed by the 5' UTR region (11.61% and 11.39%, respectively) (**Figure 3E**) We next conducted the motif prediction in the regions of m6A peak sites. **Figure 4A** shows the top five high confidential sequence motifs in the two groups. We found RRACH sequence patterns in both groups, which are typical sequence motifs proven to be related to m6A (35). Visualization of the distribution of the transcriptome-wide m6A peaks across the 20 chromosomes was performed in lung tissues. The results showed that the overlapping peaks between the two groups and the unique peaks of asthma were distributed on each chromosome, and the distribution patterns coincided with the gene content density (**Figure 4B**). Most of the hypomethylated peaks were enriched in chromosomes 2 (27peaks), 5 (23 peaks), and 7 (20 peaks), while most of the hypermethylated peaks were enriched in chromosomes 7 (76 peaks), 2 (74 peaks), and 1 (73 peaks) (**Figure 4B**). In addition, the hypomethylated peaks with the largest widths were distributed on chromosomes 15, 8, and 1, while hypermethylated peaks with the largest widths were distributed on chromosomes 16, 18, and 6 (**Figure 4B**).

### Differentially Methylated mRNAs in Asthma

**Figure 5:**
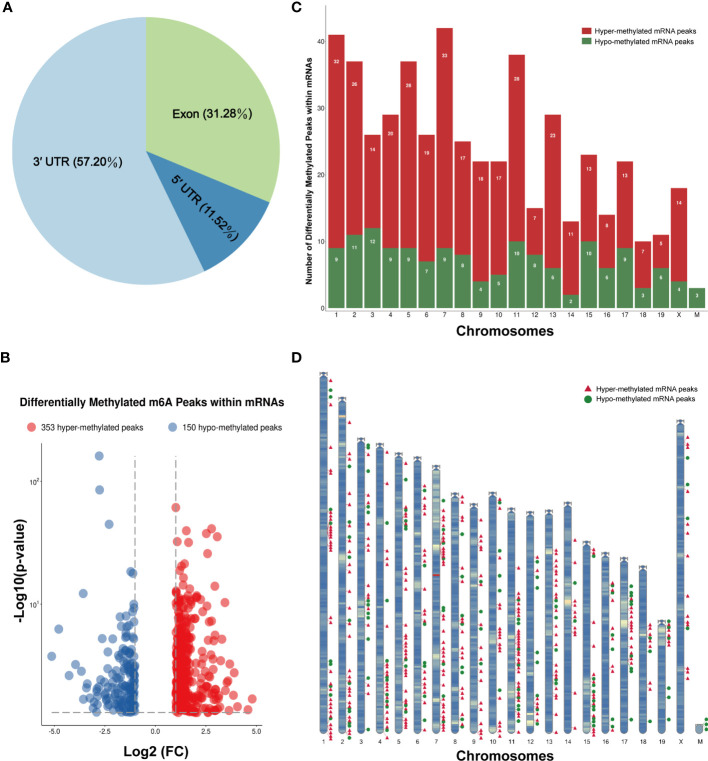
Distribution of the differentially methylated *N* ^6^-methyladenosine (m6A) peaks within mRNA transcripts. **(A)** Percentage of the differentially methylated m6A peaks in the 5' UTR, CDS, and 3' UTR regions. **(B)** The 353 hypermethylated and 150 hypomethylated m6A peaks within mRNAs were identified by comparing the m6A methylation levels of the phosphate-buffered saline (PBS) and ovalbumin (OVA) groups (|log_2_FC| > 1, *p* < 0.05). **(C, D)** Number **(C)** and chromosomal distribution patterns **(D)** of the hyper- and hypomethylated m6A peaks within mRNAs.

The m6A methylation levels of the lung tissues in the OVA and PBS groups were compared in order to explore the changes and functions of m6A methylation in asthma. We found 855 hypermethylated peaks and 300 hypomethylated peaks within whole sets of transcripts (**Supplementary Table S8**), and all differentially methylated m6A peak sites in the two groups most often appeared in the 3' UTR region (57.2%), followed by the exon (31.28%) and the 5' UTR region (11.52%) (**Figure 5A**). Furthermore, we screened out the methylated mRNA transcripts and found that there were 353 significantly hypermethylated peaks within 329 mRNA transcripts and 150 significantly hypomethylated peaks within 143 mRNA transcripts (**Figure 5B** and **Supplementary Table S9**). These mRNA transcripts containing methylated m6A peaks are hereinafter referred to as differentially methylated mRNAs. The top 20 differentially methylated mRNAs are listed in **Table 1** based on log_2_(Fold change). We calculated the density of these differentially methylated peaks in each chromosome and found that they were not distributed homogeneously (**Figure 5C**). The top five chromosomes with the largest number of peak distributions were chromosomes 7, 1, 11, 2, and 5. In detail, chromosome 7 harbored the most number of differentially methylated m6A peaks (33 hyper- and 9 hypomethylated peaks), followed by chromosome 1 (32 hyper- and 9 hypomethylated peaks), chromosome 11 (28 hyper- and 10 hypomethylated peaks), chromosome 2 (26 hyper- and 11 hypomethylated peaks), and chromosome 5 (28 hyper- and 9 hypomethylated peaks). The number of hypermethylated peaks in each chromosome was distinctly larger than that of hypomethylated peaks (apart from chromosomes 12 and 19). We also found three differentially methylated peaks in two protein-coding mitochondrial transcripts (MT-ND4 and MT-ND3), and all three peak sites were hypomethylated. MT-ND4 contained two hypomethylated peaks, while MT-ND3 contained one (**Supplementary Table S9**). Besides, we mapped the differentially methylated peaks (within mRNA transcripts) to the mouse chromosomes and visualized the peak site locations across the chromosomes using the R package RIdeogram (36) (**Figure 5D**).

**Table 1:** Top 20 differentially methylated mRNAs based on log_2_(FC).

Gene name	Chromosome	Peak start	Peak end	*p*-value	Log_2_(FC)	Regulation	Methylated region
*Postn*	chr3	54317751	54318231	2.04E−02	4.79	Hyper	3' UTR
*Cldn24*	chr8	47822372	47822517	4.17E−02	4.58	Hyper	Exon
*Lrrc66*	chr5	73607180	73607451	3.47E−02	4.06	Hyper	Exon
*Ssr1*	chr13	37967203	37967444	1.74E−03	3.90	Hyper	3' UTR
*Zfp819*	chr7	43617720	43618018	1.55E−02	3.86	Hyper	3' UTR
*Vinac1*	chr2	129038002	129039048	5.62E−04	3.68	Hyper	Exon
*Dkk2*	chr3	132085769	132085949	4.37E−02	3.64	Hyper	5' UTR
*6330403K07Rik*	chr11	71033245	71033483	4.27E−03	3.59	Hyper	5' UTR
*Mycbpap*	chr11	94521502	94521682	5.01E−03	3.54	Hyper	5' UTR
*Cetn4*	chr3	37311668	37311907	1.05E−02	3.46	Hyper	5' UTR
*Trdn*	chr10	33196064	33196305	1.74E−04	−5.13	Hypo	3' UTR
*2210418O10Rik*	chr2	176238717	176239165	5.50E−07	−4.78	Hypo	3' UTR
*Slfn14*	chr11	83275946	83276275	2.40E−03	−4.27	Hypo	3' UTR
*Bhlhe22*	chr3	18056233	18056562	6.03E−04	−3.95	Hypo	3' UTR
*Gm1043*	chr5	37174942	37175542	1.45E−03	−3.66	Hypo	Exon
*mt-Nd3*	MT	9458	9661	6.31E−13	−3.57	Hypo	Exon
*Alcam*	chr16	52453265	52453446	2.09E−02	−3.56	Hypo	5' UTR
*Ppp4r3a*	chr12	101083581	101083702	8.32E−03	−3.41	Hypo	5' UTR
*C2cd4a*	chr9	67831704	67832330	5.75E−03	−3.37	Hypo	5' UTR
*Fam122b*	X	53269684	53269805	1.91E−02	−3.24	Hypo	5' UTR

Hypo, hypomethylation; Hyper, hypermethylation.

### Functional Analysis of Differentially Methylated mRNAs

**Figure 6:**
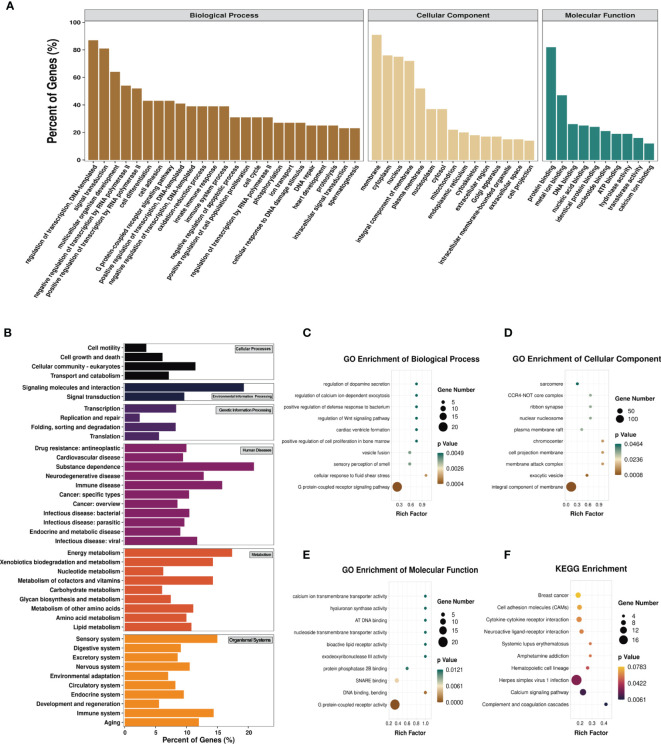
Gene Ontology (GO) function and Kyoto Encyclopedia of Genes and Genomes (KEGG) pathway analyses of the differentially methylated mRNAs. **(A)** Percentage of the differentially methylated mRNAs enriched in GO categories such as biological process (BP), cellular component (CC), and molecular function (MF). **(B)** Percentage of the differentially methylated mRNAs enriched in KEGG categories such as cellular processes, environmental information processing, genetic information processing, human diseases, metabolism, and organismal systems. **(C–F)** Top 10 significant GO terms of the BP **(C)**, CC **(D)**, and MF **(E)** categories and KEGG **(F)** pathways.

To explore the biological sense of the differentially methylated mRNAs and their potential role in the pathological processes of asthma, we performed Gene Ontology (GO) and Kyoto Encyclopedia of Genes and Genomes (KEGG) enrichment analyses. **Figure 6A** provides an overview of the distributions of the differentially methylated mRNAs enriched in various GO categories. In the biological process (BP) category, differentially methylated mRNAs were mainly enriched in “regulation of DNA-templated transcription” (87%), “signal transduction” (81%), and “multicellular organism development” (64%). In the cellular component (CC) category, the top three enriched functions were “membrane” (91%), “cytoplasm” (76%), and “nucleus” (75%). In the molecular function (MF) category, “protein binding” (82%), “metal ion binding” (47%), and “DNA binding” (26%) were the most enriched terms. We next analyzed the significant enrichment degree of the differentially methylated mRNAs in various GO terms. The differentially methylated mRNAs were found to be significantly enriched in 120 GO terms (*p* < 0.05), suggesting that they are related to diverse biological functions. For instance, the differentially methylated mRNAs were significantly enriched in “G protein-coupled receptor signaling pathway”, “sensory perception of smell”, “positive regulation of cell proliferation in bone marrow”, “regulation of Wnt signaling pathway” (BP; **Figure 6C**), “chromocenter”, “membrane attack complex”, “CCR4-NOT core complex” (CC; **Figure 6D**), “G protein-coupled receptor activity”, “calcium ion transmembrane transporter activity”, and “bioactive lipid receptor activity” (MF; **Figure 6E**). Intriguingly, we also found that these differentially methylated mRNAs were significantly enriched in GO terms related to T-cell differentiation and inflammatory response, such as “T-helper 1 cell differentiation”, “cytokine activity”, and “inflammatory response to wounding”. Detailed information on the enriched GO terms and the corresponding gene sets are shown in **Supplementary Table S10**.

Likewise, we analyzed the distributions of the differentially methylated mRNAs in the six KEGG categories. The results are shown in **Figure 6B**. In the cellular process category, the differentially methylated mRNAs were mostly distributed in “cellular community-eukaryotes” (11%) and “transport and catabolism” (7%). The most enriched pathways in the environmental information processing category were “signaling molecules and interaction” (19%) and “signal transduction” (10%). In the genetic information processing category, “transcription” (8.33%) and “folding, sorting and degradation” (8.26%) were mainly enriched. “Substance dependence” (20.93%) and “immune disease” (15.79%) were the most enriched pathways in the human disease category. In the metabolism category, the most enriched pathways were “energy metabolism” (17.39%) and “metabolism of cofactors and vitamins” (14.29%). The most enriched pathways in the organismal systems category were “sensory system” (15%) and “immune system” (14.40%). The KEGG pathways significantly enriched by the differentially methylated mRNAs were “complement and coagulation cascades”, “calcium signaling pathway”, “herpes simplex virus 1 infection”, and “hematopoietic cell lineage” (*p* < 0.05) (**Figure 6F**). Among these pathways, hematopoietic cell lineage is closely related to asthma, and the mRNAs enriched in this pathway included FCER2A, GP1BA, IL-7, and CD5. Detailed information on the enriched KEGG pathways and the corresponding gene sets are shown in **Supplementary Table S11**.

### Prediction of RNA-Binding Proteins

**Figure 7:**
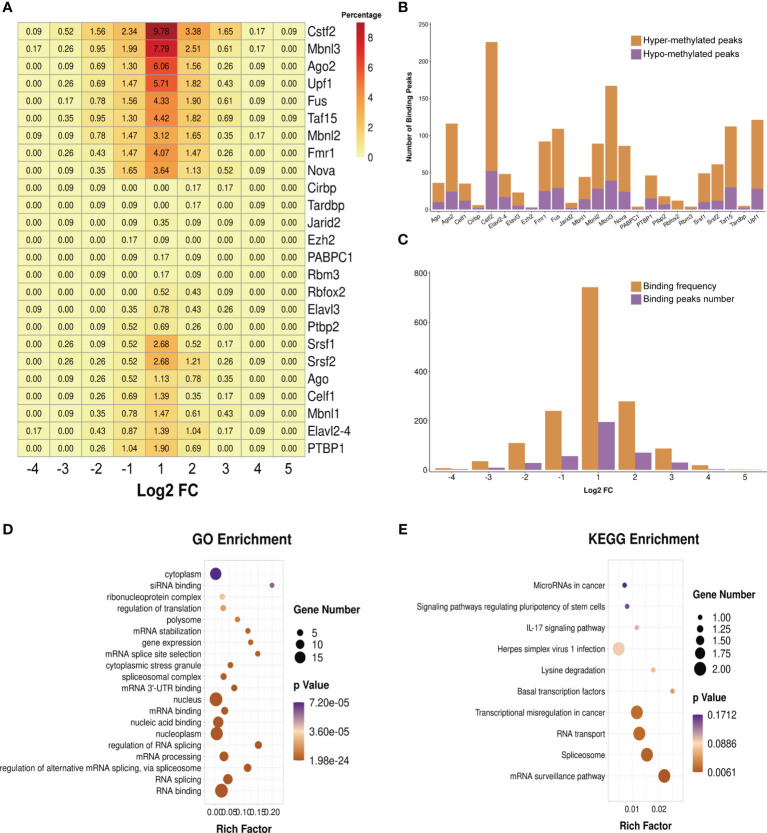
Prediction and function of RNA-binding proteins (RBPs). **(A)** Heatmap showing the binding rates of the 25 RBPs. Values are presented as the binding percentage of RBPs in the total differentially methylated peaks. **(B)** Number of hyper- and hypomethylated peaks bound by RBPs. **(C)** Distribution of RBPs in differentially methylated peaks with different fold change values. **(D, E)** Gene Ontology (GO) **(D)** and Kyoto Encyclopedia of Genes and Genomes (KEGG) **(E)** enrichment analyses of the 25 predicted RBP genes.

We predicted the RBPs that might bind to the differentially methylated peaks within the whole sets of transcripts *via* exploring the RMBase (v2.0) database (37). The 25 potential RBPs in the 299 hypermethylated peaks and 24 potential RBPs in the 96 hypomethylated peaks were obtained. We classified these bound peaks into nine groups according to log2(FC). **Figure 7A** shows the binding percentage of RBPs in the total differentially methylated m6A peaks. The results displayed that the binding frequency of Cstf2, Mbnl3, Upf1, Ago2, Taf15, Fus, Fmr1, Mbnl2, and Nova in the peaks of each group shared similar distribution patterns. The RBPs which bound the most of peaks were Cstf2 (bound 226 peaks), Mbnl3 (bound 167 peaks), Upf1 (bound 121 peaks), Ago2 (bound 116 peaks), Taf15 (bound 112 peaks), and Fus (bound 109 peaks). The binding abundance of RBPs in the hypermethylated peaks is higher than that in the hypomethylated peaks (**Figure 7B**), which indicated that these RBPs prefer to target hypermethylated peaks. Additionally, the RBPs and their binding peaks were mostly distributed in the group of differentially methylated peaks with log_2_(Fold change) = 1 (**Figure 7C**).

We then performed GO and KEGG enrichment analyses on these 25 RBPs, and the results revealed that RBPs were significantly enriched in GO terms and KEGG pathways related to the biogenesis and metabolism of RNA. For instance, RBPs were significantly enriched in the GO terms “RNA splicing”, “regulation of alternative mRNA splicing *via* spliceosome”, “mRNA processing”, “regulation of RNA splicing”, “mRNA 3' UTR binding”, “spliceosomal complex”, “mRNA splice site selection”, and “mRNA stabilization” (**Figure 7D**). The results of KEGG enrichment showed that these RBPs were significantly enriched in “mRNA surveillance pathway”, “spliceosome”, and “RNA transport” (**Figure 7E**).

### Differentially Expressed Genes in Asthma

**Figure 8:**
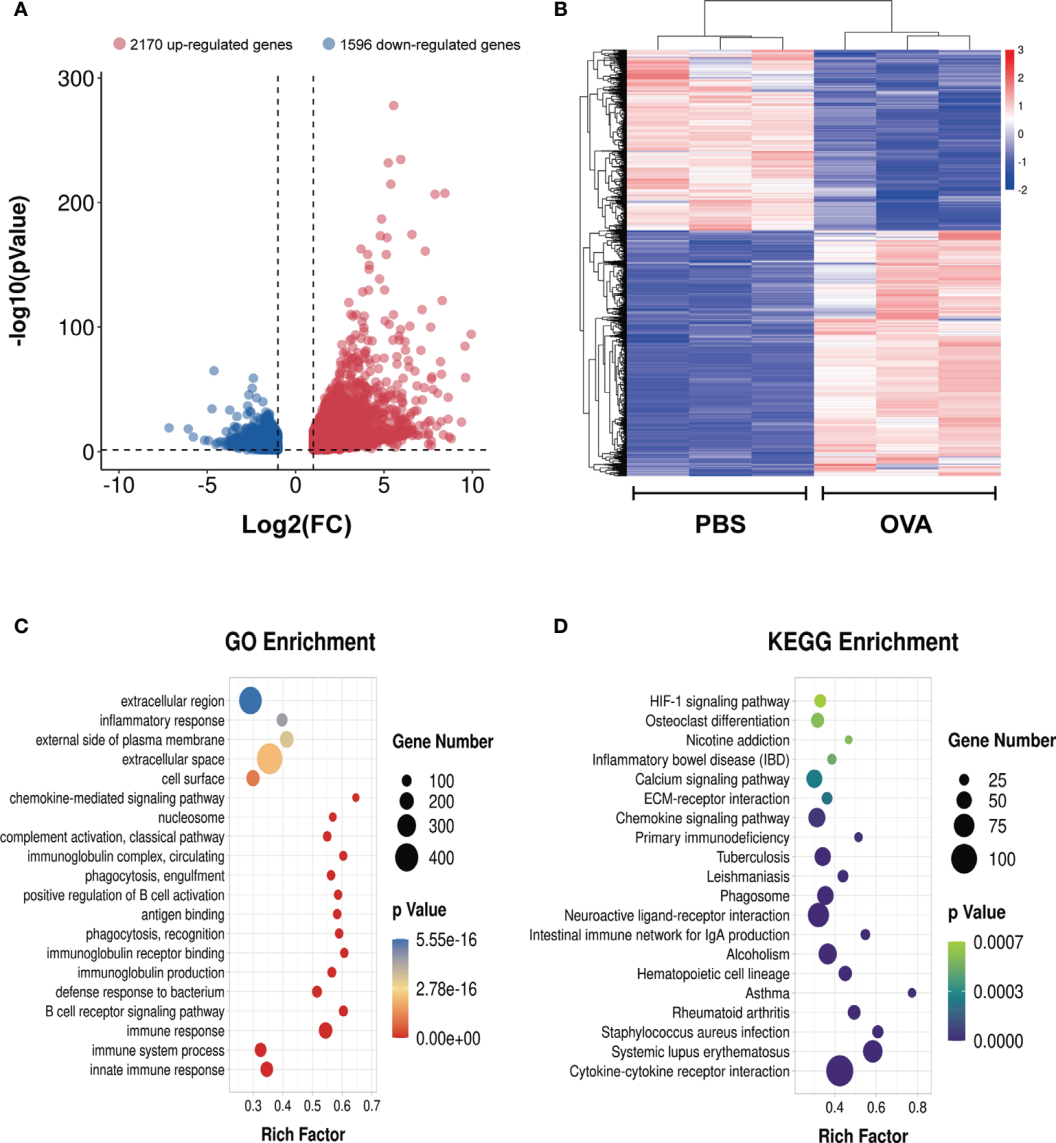
Differentially expressed genes in the lung tissues of ovalbumin (OVA)-induced asthma. Volcano plot **(A)** and heatmap **(B)** showing the significantly differentially expressed genes in lung tissues (|log_2_ FC| > 1, *p* < 0.05). Gene Ontology (GO) **(C)** and Kyoto Encyclopedia of Genes and Genomes (KEGG) **(D)** enrichment analyses of the differentially expressed genes.

To identify the differential gene expression profiles in asthmatic lung tissues, we compared the differential gene expressions of the OVA and PBS groups and found a total of 3,766 differentially expressed genes (**Supplementary Table S12**), among which 2,170 were upregulated and 1,596 were downregulated in asthma (**Figure 8A**). A similar trend of expression was displayed in the same group of lung tissues regarding the differentially expressed genes (**Figure 8B**). We further conducted GO and KEGG enrichment analyses for these differentially expressed genes. The results of GO enrichment showed that the function of these genes was involved in immune response and regulation, including “immune system process”, “immune response”, “antigen binding”, “positive regulation of B-cell activation”, and “chemokine-mediated signaling pathway” (**Figure 8C**). KEGG analysis identified a bunch of significantly enriched pathways related to immune regulation and diseases, such as “cytokine–cytokine receptor interaction”, “chemokine signaling pathway”, “HIF-1 signaling pathway”, “systemic lupus erythematosus”, “rheumatoid arthritis”, and “asthma” (**Figure 8D**).

### Joint Profiling of m6A Methylation and Gene Expression in Asthmatic Lung Tissues

**Figure 9:**
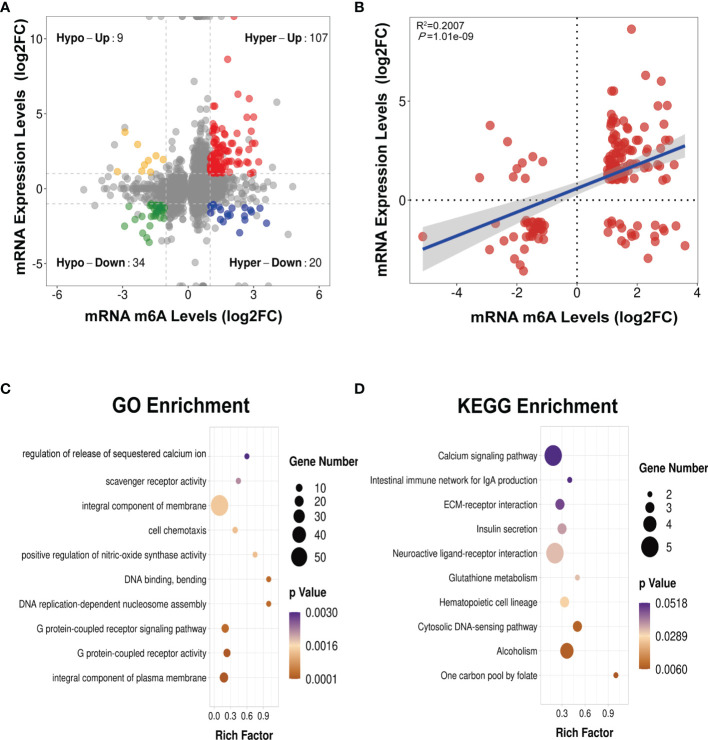
Conjoint analysis of MeRIP-seq and RNA-seq data **(A)** Four-quadrant plot showing the differentially methylated peaks within differentially expressed mRNAs (|log_2_ FC> 1, *p* < 0.05). **(B)** Positive correlation between the *N* ^6^-methyladenosine (m6A) levels and the expression levels of mRNAs with both differential methylation and expression (*R* ^2^ = 0.2007, *p* = 1.01e−09). **(C, D)** Gene Ontology (GO) **(C)** and Kyoto Encyclopedia of Genes and Genomes (KEGG) **(D)** enrichment analyses of mRNAs with both differential methylation and expression. *Hypo*, hypomethylation; *Hyper*, hypermethylation; *Up*, upregulated expression; *Down*, downregulated expression.

We next conducted a combined analysis of the MeRIP- and RNA-seq data. Significant differential expressions were found in 170 differentially methylated mRNA transcripts (**Figure 9A** and **Supplementary Table S13**). Specifically, from the 127 hypermethylated mRNAs, we obtained 107 highly and 20 low expressed mRNAs. From the 43 hypomethylated mRNAs, we obtained nine highly expressed and 34 low expressed mRNAs (**Figure 9A**). The top 20 differently methylated mRNAs with differential expressions are shown in **Table 2**. To clarify the relationship between the methylation levels and the expression levels of these 170 mRNAs, we further performed Pearson’s correlation analysis and found that the methylation levels of these mRNAs were positively correlated with their expression levels (*R* ^2^ = 0.2007, *P* = 1.01e−9) (**Figure 9B**). This suggested that m6A methylation plays a crucial role in the regulation of gene expression in asthma.

**Table 2:** The top 20 differentially methylated mRNAs with differential expression based on log_2_(FC) of gene expression.

Gene name	Chromosome	Methylation regulation			Expression regulation		
		Log_2_(FC)	*p*-value	Regulation	Log_2_(FC)	*p*-value	Regulation
*Cebpe*	chr14	2.10	1.55E−02	Hyper	inf	2.25E−35	Up
*Msx3*	chr7	1.81	3.98E−02	Hyper	8.63	2.99E−44	Up
*Syt13*	chr2	2.28	3.16E−03	Hyper	6.30	9.28E−16	Up
*Gpr84*	chr15	2.80	1.41E−02	Hyper	6.01	5.83E−10	Up
*Spp1*	chr5	1.22	8.32E−06	Hyper	5.51	2.45E−90	Up
*Hrc*	chr7	2.36	3.89E−02	Hyper	−2.94	1.78E−09	Down
*6330403K07Rik*	chr11	3.59	4.27E−03	Hyper	−2.30	1.98E−05	Down
*Perm1*	chr4	1.61	2.00E−12	Hyper	−2.21	1.79E−05	Down
*Paqr9*	chr9	2.60	7.76E−03	Hyper	−2.09	7.91E−06	Down
*Gp1ba*	chr11	1.48	1.07E−02	Hyper	−2.06	7.39E−06	Down
*Clspn*	chr4	−2.89	9.12E−03	Hypo	3.77	3.04E−36	Up
*Msc*	chr1	−2.31	1.95E−02	Hypo	2.95	2.29E−10	Up
*Hdac9*	chr12	−1.47	2.00E−02	Hypo	2.19	6.36E−31	Up
*Gm7609*	chr1	−1.14	2.69E−02	Hypo	1.94	1.09E−05	Up
*Cpz*	chr5	−1.82	3.47E−03	Hypo	1.88	1.37E−06	Up
*Fpr1*	chr17	−1.78	2.24E−04	Hypo	−3.58	2.24E−17	Down
*Tcap*	chr11	−1.89	2.24E−02	Hypo	−3.28	1.84E−08	Down
*Xirp2*	chr2	−2.08	4.27E−02	Hypo	−2.97	2.50E−10	Down
*Rbm20*	chr19	−2.91	4.79E−02	Hypo	−2.50	1.31E−05	Down
*Omd*	chr13	−1.75	1.35E−08	Hypo	−2.48	6.06E−05	Down

Hypo, hypomethylation; Hyper, hypermethylation; Up, upregulated expression; Down, downregulated expression.

**Figure 10:**
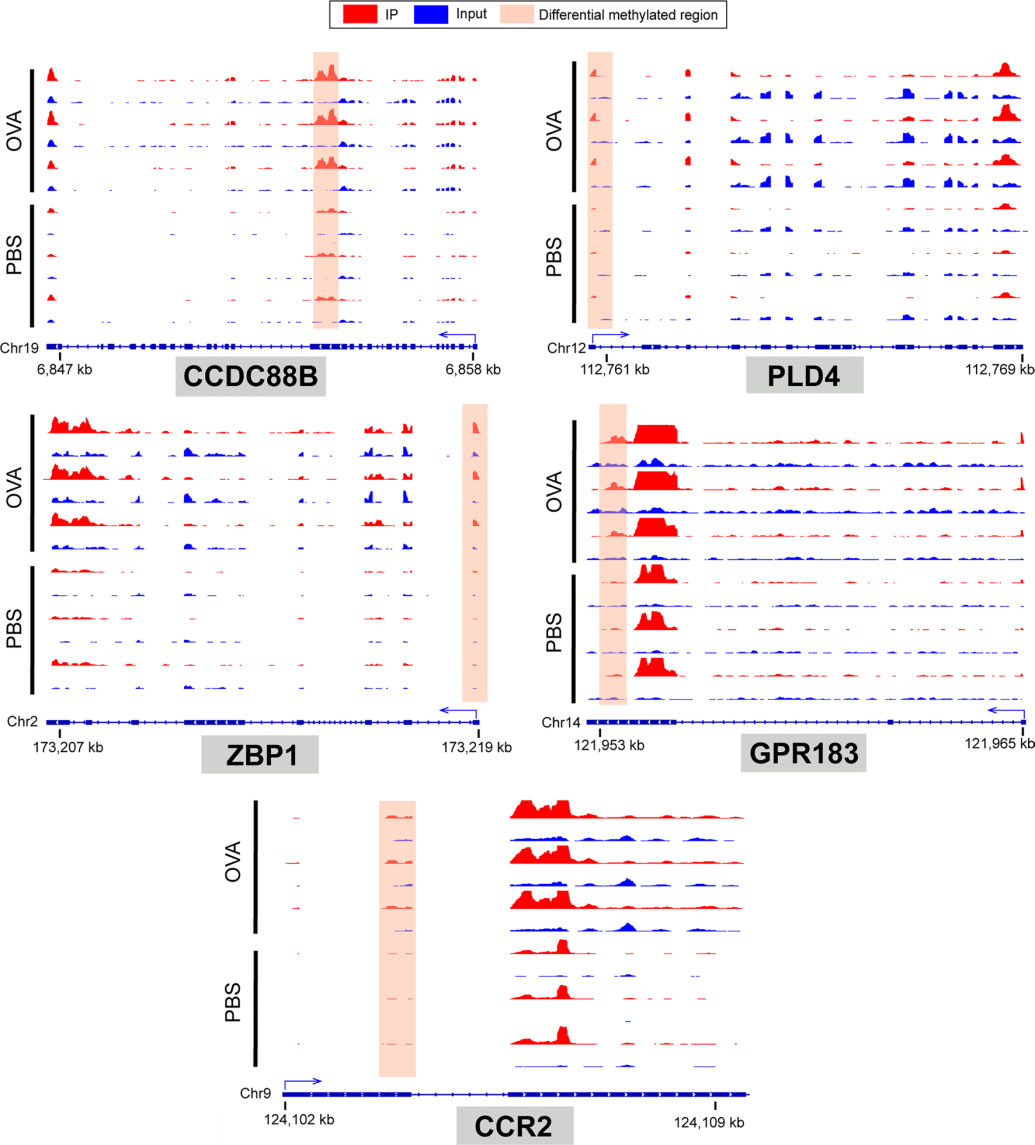
IGV tracks display the region distribution of peaks in CCDC88B, PLD4, ZBP1, GPR183, CCR2 genes. The peaks are located in the exon region of CCDC88B; 5' UTR region of PLD4, ZPB1 and CCR2; 3' UTR regions of GPR183.

To identify the roles of these differentially methylated mRNAs with expression changes in asthma, we performed GO and KEGG enrichment analyses. For the GO enrichment results, the GO terms most significantly enriched were “integral component of plasma membrane”, “G protein-coupled receptor activity”, and “G protein-coupled receptor signaling pathway” (**Figure 9C**). We also found that many GO terms related to immune function and inflammatory response were significantly enriched, such as “inflammatory response”, “innate immune response”, “positive regulation of T-cell activation”, “positive regulation of cytokine production”, “response to interleukin-1”, “chemokine receptor activity”, and “T-cell chemotaxis” (**Supplementary Table S14**), which indicated that m6A methylation may have an immunoregulatory effect in asthma. The KEGG enrichment results showed that these differentially methylated mRNAs with expression changes were significantly enriched in “one carbon pool by folate”, “alcoholism”, “cytosolic DNA-sensing pathway”, “hematopoietic cell lineage”, among others (**Figure 9D** and **Supplementary Table S15**). Given that the pathological mechanism of asthma involves immune dysregulation and excessive inflammation, we thus explored whether there are differential m6A-modified mRNAs associated with pathological processes. Interestingly, a bunch of differentially methylated mRNAs related to immune regulation were found. Here, we enumerated five representative mRNAs (*CCDC88B*, *PLD4*, *ZBP1*, *GPR183*, and *CCR2*) and visualized the distribution of the peak regions in these mRNAs using IGV software (**Figure 10**).

## Discussion

### Progress of m6A Methylation in Asthma

The m6A methylation of RNA is a reversible epigenetic modification (38), and its methylation level is tightly regulated by m6A methylase and demethylase. m6A modification is mediated and facilitated by methylases such as METTL3 (methyltransferase-like 3), METTL14, and WTAP (Wilms’ tumor 1-associated protein), whereas modification can be reversed by demethylases such as FTO (fat mass and obesity-associated protein) and ALKBH5 (alkylation repair homolog 5) (35). Thus far, only two studies have directly investigated the relationship between asthma and m6A modification. One of them showed that m6A enzymes may have a role in the pathogenesis of asthma through bioinformatics analysis of the public dataset from the GEO database (39). This study analyzed the gene expressions of m6A enzymes in the peripheral blood mononuclear cells of children (average age, 8.4 years) with asthma and found two different expression patterns of the m6A enzymes that were associated with the immune responses of T helper cells (Th1 and Th2). Besides, the degree of immune infiltration was higher in patients with an upregulated methylase WTAP gene expression compared to that in patients with a downregulated expression (39). Another intensive research reported on the potential mechanism of m6A modification in the Th2 immune response of asthma (40). The research group found that OVA-induced FTO knockout mice exhibited evident defects of motile ciliogenesis and asthma-like inflammatory phenotypes, such as the upregulated expression of the Th2 cytokine (IL-13) and the downregulated expression of the Th1 cytokine (IL-12b) mRNA in lung tissues, and significantly increased levels of IL-13 and IL-4 in BALF (40). They speculated that the decrease of ciliary cells in the airway epithelium may be responsible for the aggravation of airway Th2 immune response.

Dysfunction of airway epithelial cells underlies the pathogenesis of airway hyperresponsiveness and inflammation in asthma. A recent study investigated the role of m6A modification in human airway epithelial cells (41). The results showed that the expression level of methylase METTL3 mRNA and the m6A modification level of total RNA were significantly increased in the human bronchial epithelial (HBE) and A549 cells induced by fine particulate matter (PM_2.5_). Subsequent findings suggested that the METTL3 protein could maintain the stability of the oxidative stress-induced growth inhibitor 1 (*OSGIN1*) mRNA by increasing the m6A methylation level and thus participate in PM_2.5_-induced airway epithelial cell damage (41). Likewise, another recent study examined the role of the demethylase FTO in the differentiation of HBE cells to ciliated cells (40). The results showed that the mRNA levels of *FTO* and master ciliary transcription factor (*FOXJ1*) were significantly increased in epithelial cells cultured under the condition of air–liquid interface after 14 days. The protein levels of *FTO* and *FOXJ1* showed the same trend after 16 days of culture. In addition, *FTO* knockdown HBE cells exhibited inhibition of the expression of *FOXJ1* and differentiation of ciliated cells; the goblet cell numbers were instead increased (40). The researchers further observed that the multi-ciliated cells and the *FOXJ1* mRNA levels in the lung tissues of FTO knockout mice were lower than those in wild-type mice. These findings revealed that m6A demethylation may affect the formation of motor cilia in the airway epithelium by regulating the expression of *FOXJ1*.

However, no previous study has comprehensively examined the m6A modification characteristics in strictly identified animal models of asthma or patients with asthma. The above studies neither explored the mechanism of m6A modification that contributed to the asthmatic immune response nor the role of m6A modification in the most important phenotype of asthma, airway hyperreactivity. To complement these studies, we therefore employed MeRIP-seq and RNA-seq to reveal, for the first time, the m6A methylation characteristics of asthmatic mice at the whole transcriptome level and to analyze in detail the asthma-related methylated genes and their potential functions.

### Asthmatic Phenotypes Induced by OVA

Airway inflammation and hyperreactivity are two typical pathological hallmarks of asthma (42). The inflammatory process of asthma is characterized by excessive Th2 immune response caused by the imbalance of the Th1/Th2 cell ratio. The increased Th2 cells subsequently secrete large quantities of cytokines such as IL-4, IL-5, and IL-13. IL-4 promotes the differentiation of memory B cells, which produce a plethora of IgE when stimulated by antigens. IL-5 induces the infiltration of eosinophilic cells into lung tissues, and IL-13 activates a vast panel of inflammatory cells and promotes mucus secretion and bronchial constriction (43). IgE and Th2-type cytokines further aggravate airway hyperreactivity and elicit airway remodeling by activating fibroblasts (28). In the OVA-induced mice, we observed typical airway hyperresponsiveness and reduced pulmonary compliance, which represent the most important indicators to evaluate the success of the establishment of a mouse model of asthma. We further detected that the OVA-induced mice presented excessive Th2-type immune response; for instance, the levels of IL-4, IL-5, and IL-13 in serum and IgE in BALF were significantly increased. Moreover, the number and percentage of eosinophils in the BALF of the model group were significantly higher than those in the control group; so were the number of total and different subtypes of white blood cells. HE, PAS, and MT staining respectively displayed massive infiltration of inflammatory cells, mucus hypersecretion, and airway structure change in lung tissues. Overall, these results validated that OVA successfully induced airway hyperresponsiveness and inflammation, goblet cell hyperplasia, and airway remodeling, which conform to the pathological changes of asthma (44, 45), thus demonstrating the effectiveness of our model.

### The m6A Methylation Pattern in Asthma

Our results showed that there were prominent m6A modifications in both normal and OVA-induced lung tissues. The two groups have approximately the same distribution characteristics of the m6A peaks, such as the peak frequency and width within mRNA transcripts and the distribution of m6A peaks in genic regions. Nevertheless, there were a large number of unique m6A peaks and methylated mRNAs in OVA-induced lung tissues. We speculated that OVA induced a specific pattern of m6A methylated modification. m6A modification was widely distributed in the RNA transcripts of eukaryotes, and the m6A peaks were highly abundant in different organs and tissues. In this study, we identified 27,292 m6A peaks in OVA-induced lung tissues. Most of these m6A peaks were distributed in the 3' UTR and exon regions, which is inconsistent with the previously reported peak distribution trend in mouse liver (46), sheep liver (47), rat heart (48), and human tumor tissue (49, 50). We considered that such inconsistencies may be related to different species, tissues, and conditions. In the OVA-induced lung tissues, we also found various subsets of the typical sequence motif RRACH which have been confirmed to be closely related to the enrichment of m6A peaks (35). Our results demonstrated that m6A methylation might have a role in asthma disease.

### Differential Methylated mRNAs Contribute to Airway Inflammation in Asthma

We analyzed the distribution of the differentially methylated peaks in the mRNA transcripts and found that there were 353 significantly hypermethylated and 150 significantly hypomethylated m6A peaks within 329 and 143 mRNA transcripts, respectively. To determine the biological functions of the differentially methylated mRNAs in asthma, we performed GO and KEGG enrichment analyses. The GO enrichment results showed that these differentially methylated mRNAs were significantly enriched in 120 GO terms. Of these, “regulation of Wnt signaling pathway”, “T-helper 1 cell differentiation”, “G protein-coupled receptor signaling pathway”, “G protein-coupled receptor activity”, and “cytokine activity” were closely related to immune function. Several previous studies have found that the Wnt signaling pathway have immunomodulatory effects and are closely related to lung function in asthma. For instance, the Wnt signaling pathway has been shown to be positively correlated with the airway Th2 immune response in asthmatic patients (51, 52). The Wnt/β-catenin pathway combined with transforming growth factor beta (TGF-β) can regulate airway remodeling mediated by epithelial–mesenchymal transition in asthma patients (53). Wnt1 was able to reduce airway allergic inflammation and the hyperresponsiveness of asthma by inhibiting the activation of pulmonary dendritic cells (54). Activation of Wnt-3A promoted the maturation of mast cells, thereby further aggravating the infiltration of immune cells in asthmatic airways (52). Th1 cells are considered to inhibit the function of Th2 cells and thus play an anti-asthmatic role (55). The overreaction of Th2 cells led to the imbalance of the Th1/Th2 ratio, which may increase exacerbations in asthma (56). In contrast, several researches have verified that Th1 cells promoted airway neutrophil infiltration and Th2 cell-induced eosinophil recruitment. Both Th1 and Th2 cells were able to induce airway inflammation. Moreover, the airway hyperresponsiveness induced by Th1 cells was even more severe than that induced by Th2 cells (57). A study has shown *in vitro* that OVA, IL-2, and IL-18 could stimulate Th1 cells to produce a vast panel of cytokines such as IFN-γ, IL-9, IL-13, GM-CSF, RANTES, and MIP-1. *In vivo* experiments have suggested that intranasal interventions with OVA and IL-18 in mice receiving adoptive transfer of Th1 cells could induce severe airway inflammation and hyperresponsiveness (58).

Differentially methylated mRNAs were enriched on the G protein-coupled receptor (GPR)-related GO terms. Multiple studies have confirmed that many GPR family molecules were involved in the functional regulation of immune cells. For example, lysophosphatidylserine suppresses the IL-2 secretion of CD4^+^ T cells through GPR174 (59). GPR174 inhibited the proliferation of regulator T cells in the thymus (60), and GPR84 signaling could promote macrophages to produce inflammatory cytokines and chemokines, such as TNF-α, IL-6, IL-12b, CCL2, CCL5, and CXCL1 (61). Lipopolysaccharide (LPS) induced GPR84 expression in monocytes/macrophages, and GPR84, in turn, promoted IL-12b secretion by monocytes/macrophages (62), which further facilitated the differentiation of Th1 cells and the generation of related cytokines, thus affecting the balance of Th1/Th2 (63). Medium-chain free fatty acids regulated the balance of Th1/Th2 by directly acting on GPR84 (62), and GPR21 can promote the migration of macrophages to inflammatory tissues (64). In addition, the differentially methylated gene *CXCL16* enriched in the GO term “cytokine activity” could enhance Th1-type immune response (65).

The KEGG enrichment results showed that the differentially methylated mRNAs significantly enriched in the pathway “hematopoietic cell lineage” were *FCER2A*, *GP1BA*, *IL-7*, and *CD5*. Hematopoietic stem cells are well-known progenitors that can differentiate into multiple essential immune cells, including T cells, B cells, natural killer cells, dendritic cells, and macrophages (66). The differentially methylated mRNAs enriched in this pathway were involved in the regulation of immune function. For example, *FCER2A* (CD23) is associated with the activation of B cells (67). *GP1BA*, a glycoprotein component on the surface of platelets is often related to clotting function (68) and has also been considered to participate in lung inflammation (69, 70). IL-7 is considered to activate eosinophils and has a pivotal function in allergic inflammation in asthma (71), but is also essential for T- and B-cell development (72). CD5, a transmembrane protein, is expressed in both T and B cells that affects thymocyte development and negatively regulates T-cell activation (73). In our case, we speculated that these differentially methylated mRNAs may be playing a role in the pathogenesis of asthma by regulating the functions of immune cells. Additionally, these differentially methylated mRNAs were also significantly enriched in pathways including “complement and coagulation cascades”, “calcium signaling pathway”, and “herpes simplex virus 1 infection”. Although these pathways have not been reported in asthma, they deserve attention.

### RNA-Binding Proteins in Asthma

RBPs have substantial implications in gene expression and the occurrence and progression of diseases. RBPs affect RNA fate through binding to RNAs for regulatory alternative splicing, nuclear export, stability, localization, and translation (74). The m6A sites in RNA have been found to be specifically recognized by a number of RBPs, such as the YTH domain family proteins and HNRNP family proteins (35), which are the executors of m6A-associated biological functions. Due to certain diverse RBPs that can bind to m6A peak sites, the regulation of RNA processes by m6A methylation is highly heterogeneous (75). Interestingly, recent research has reported that the RBPs were also directly involved in the pathogenesis of asthma. The researchers adopted the method of OVA combined with complete Freund’s adjuvant induction to establish refractory neutrophilic inflammation of asthma. They observed that the RBP Mex-3B knockout mice have less neutrophilic airway inflammation and airway responsiveness than did wild-type mice (76). To identify how differentially methylated mRNAs function, we explored the RMBase (v2.0) database (37) to predict the potential RBPs that may bind to the methylated peak sites. Subsequent results showed that 395 differentially methylated sites were bound by 25 RBPs in asthmatic lung tissues. Accordingly, a study also predicted 25 RBPs that bound to 163 differentially methylated sites in liver tissues of high-fat diet-induced mice (46). This hinted at the distribution of RBPs among different tissues being conserved. In order to identify the functions of these RBPs in asthmatic lung tissues, we conducted GO and KEGG enrichment analyses. The results showed that the functions of these RBPs prevailed in mRNA biogenesis and metabolism, such as “mRNA processing”, “regulation of RNA splicing”, “mRNA binding”, “mRNA splice site selection”, “mRNA stabilization”, “mRNA surveillance pathway”, and “RNA transport”. We speculated that RBPs may affect the expressions of these differentially methylated transcripts by regulating RNA processes, thus participating in the pathological mechanism of asthma, which is worth further exploration in a future study.

### m6A Methylation Regulates Gene Expression

m6A methylation has been reported to affect mRNA stability and degradation and relies on the binding of m6A-related RBPs, thereby regulating mRNA expression (2, 38). To investigate whether m6A methylation affects gene expression in asthma, we assessed the expressions of genes in the differentially methylated sites. Firstly, we analyzed the gene expression profiles of asthmatic lung tissues and obtained 2,170 upregulated and 1,596 downregulated genes. These differentially expressed genes were mostly related to the immune regulatory mechanism of asthma, such as “immune system process”, “innate immune response”, “B-cell receptor signaling pathway”, “immunoglobulin production”, “immunoglobulin receptor binding”, “phagocytosis”, “recognition”, “antigen binding”, “chemokine signaling pathway”, and “cytokine–cytokine receptor interaction”.

Next, we proceeded to jointly analyze the differentially expressed genes and differentially methylated peaks in asthmatic lung tissues and obtained 107 hypermethylated mRNAs with high expression, 20 hypermethylated mRNAs with low expression, 9 hypomethylated mRNAs with high expression, and 34 hypomethylated mRNAs with low expression. In addition, Pearson’s correlation analysis showed that the methylation levels of these genes were positively correlated with their expression levels. In our results, the hypermethylated mRNAs with high expression accounted for the largest proportion in asthmatic lung tissues. We concluded that, in asthma, the effect of m6A methylation in maintaining the stability of mRNAs predominates. However, other differential mRNAs, such as hypermethylated mRNAs with low expression and hypomethylated mRNAs with differential expression, may also play important roles in asthma, although they accounted for only low proportion. In this case, which m6A-related RBPs regulated these differentially methylated mRNAs with differential expression and how these mRNAs play a role in asthma will be further investigated in the future.

Furthermore, the results of the enrichment analyses showed that a portion of these mRNAs was associated with immune function such as “inflammatory response”, “innate immune response”, “positive regulation of T-cell activation”, “positive regulation of cytokine production”, “response to interleukin-1”, “chemokine receptor activity”, “T-cell chemotaxis”, and “hematopoietic cell lineage”. We selected five representative differentially methylated mRNAs with differential expressions (*CCDC88B*, *PLD4*, *ZBP1*, *GPR183*, and *CCR2*) from the above pathways and reviewed their roles in immunity. *CCDC88B* has a critical impact on the regulation of T-cell function. It has been reported that *CCDC88B* was highly expressed in CD4^+^ and CD8^+^ T cells and could regulate the maturation, activation, and proliferation of T cells and the production of related cytokines such as IFN-γ and TNF (77). In addition, *CCDC88B* was also involved in the occurrence of inflammatory diseases such as colitis (78). For example, *CCDC88B* expression was significantly elevated in lymphatic and myeloid cells of the colon lamina propria in patients with ulcerative colitis and Crohn’s disease. Similarly, *CCDC88B*-positive lymphocytes and myeloid cells were distinctly increased in the inflammatory tissues from a dextran sodium sulfate-induced mouse colitis model. Further research has shown that the deficiency of *CCDC88B* in immune cells can markedly diminish the inflammatory injury of the intestinal epithelial barrier and play an anti-colitis role (78). *PLD4* is closely associated with auto-inflammatory and autoimmune conditions (79). A previous study reported on the inflammatory phenotypes in *PLD4*-deficient mice, such as splenomegaly, and increased concentrations of IFN-γ and CXCL10 in plasma. Dendritic cells in *PLD4*-deficient mice exhibited enhanced TLR9-mediated inflammatory responses (80). PLD4 is considered to be a 5' exonuclease that can degrade the ligand of TLR9, thereby reduce TLR9-mediated inflammatory response. When both *PLD3* and *PLD4* genes are lacking, neonatal mice developed severe liver inflammation with extensive infiltration of CD68^+^ myeloid cells and exhibited significantly increased levels of cytokines (IL-6, IL-10, TNF, and IFN-γ) and chemokines (CXCL10, CCL7, and CCL2) in serum (80). Besides, *PDL4* is also believed to play a regulatory role in B-cell activation (81). ZBP1 is a key inflammatory mediator (82) and an innate immune sensor, which can regulate programmed cell death and inflammatory response (83). For instance, ZBP1 can mediate the necrosis or apoptosis of macrophages and induce the generation of inflammasome, thereby promoting the production of IL-1β. ZBP1 also elicited the activation of the classical inflammatory signaling pathway NF-κB (84) and may be involved in IL-17-mediated immune responses (85). The G-protein-coupled receptor GPR183 was highly expressed in immune cells and played an important role in adaptive immune response (86). GPR183 can not only promote the migration of CD4^+^ T cells to the outer T region and the differentiation of T follicular helper cells (87) but also control the migration of B cells and dendritic cells and the production of T-cell-dependent antibodies (88, 89). CCR2 belongs to a type of chemokine receptors widely expressed on monocytes, macrophages, Th1, natural killer cells, basophils, and immature dendritic cells and participates in monocyte migration and Th1-type adaptive immunity (90, 91). Evidence suggests that CCR2 can promote the release of monocytes from bone marrow into peripheral circulation and mediate the further migration of monocytes to local inflammatory tissues (92). CCR2 also plays a significant part in Th2-type immune response. For example, CCR2 knockout mice induced by diesel exhaust particles displayed complete abolishment of the recruitment of monocytes and dendritic cells in lung tissues and the disappearance of Th2 response in mediastinal lymph nodes (93). Otherwise, it has been reported that both Th1 and Th2 immune responses can promote the mRNA expression of *CCR2* chemokine ligands CCL2, CCL7, and CCL12 in skin tissues (94).

Based on all of the above findings, we hypothesize that the methylation alterations, by affecting the RNA life cycle *via* RBPs, can regulate mRNA expression and function in immune cells, thereby participating in the allergic immune response of asthma.

### Conclusions and Future Perspectives

Taken together, we identified a list of differentially methylated mRNAs and their potential binding proteins, which may serve as vital regulators in the pathogenesis of asthma. To our knowledge, we mapped the first mouse m6A methylomic landscape in the lung tissues following OVA-induced asthma, and our results provided a novel direction for understanding the mechanism of m6A methylation affecting allergic inflammation and screening potential therapeutic targets in asthma.

However, in this study, the expressions of the m6A-related enzymes and RBPs in the lung tissues of asthmatic mice were not examined. The interaction between the differentially methylated genes and RBPs and the mechanism by which RBPs regulate the expression levels of differentially methylated genes in asthma will be the focus of future research. Moreover, the roles of the differentially methylated mRNAs and the m6A-related enzymes and RBPs in asthmatic phenotypes such as airway inflammation, hyperresponsiveness, and remodeling deserve further investigation.

## Materials and Methods

### OVA-Induced Acute Allergic Asthma Model

Female BALB/c mice (6–8 weeks old) were raised and maintained at the Animal Center of Fudan University School of Pharmacy under specific pathogen-free conditions with food and water *ad libitum*. The animal study protocol was reviewed and approved by the Experimental Animal Ethics Committee of Fudan University School of Pharmacy (approval no. 2020-12-HSYY-WY-01). The mouse model of acute allergic asthma was prepared based on the procedures reported in a previous research, with slight modifications (95). The schematic design of the animal study is shown in **Figure 1A**. In detail, mice were randomly assigned to the normal control group (PBS, *n* = 12) and the asthma group (OVA, *n* = 12). For allergy sensitization, on days 0 and 7, each mouse was intraperitoneally injected with 20 μg/ml OVA (grade V, Sigma-Aldrich Inc., St. Louis, MO, USA) and 2 mg/ml aluminum hydroxide adjuvants (Sigma-Aldrich Shanghai Trading Co., Ltd., Shanghai, China) in 0.2 ml PBS solution or with 0.2 ml PBS alone as a control. For allergen challenge, from days 14 to 20, mice were exposed to 3% OVA (grade II, Sigma-Aldrich Inc., St. Louis, MO, USA) in PBS solution aerosolized *via* ultrasonic nebulizer (Yuyue Medical Equipment Co., Ltd., Shanghai, China) once daily for 30 min or exposed to PBS as a control.

### Bronchial Provocation Test

A bronchial provocation test was performed to determine airway hyperresponsiveness in mice. Here, we adopted methacholine (Sigma-Aldrich Shanghai Trading Co., Ltd., Shanghai, China) as the stimulus drug. One day after the last OVA challenge (day 21), airway resistance and dynamic compliance (*C*_dyn_) in mice were evaluated using the FinePointe RC System (DSI Buxco Electronics, Troy, NY, USA). Specific details of the measurement were described in our previous study (44, 45). Briefly, mice were intubated by tracheotomy under anesthesia and ventilated with 2 μl nebulized methacholine of different concentrations (0, 3.125, 12.5, and 50 mg/ml). The mouse bronchus was sequentially provoked with methacholine of increasing concentrations and the extrema of airway resistance and *C*_dyn_ recorded for each provocation. After the bronchial provocation test, whole blood was collected from anesthetized mice *via* quickly removing an eyeball. Mice were subsequently sacrificed by cervical dislocation under anesthetic status. Then, the BALF and lung tissues were collected and subjected to subsequent assays.

### Analysis of IgE, Cytokines, and Leukocytes

Whole blood was centrifuged (3,000 rpm, 30 min) and serum was separated for detecting the level of IgE. BALF was centrifuged (500 × *g*, 10 min) and the resultant supernatant was used for measuring the levels of IL-4, IL-5, and IL-13. These immune and inflammation-related protein levels were measured using ELISA following instructions in the kit (MultiSciences Biotech Co., Ltd., Hangzhou, China). The precipitate from BALF was collected to calculate the numbers and percentages of the different subsets of leukocytes using Auto Hematology Analyzer (Shenzhen Mindray Bio-Medical Electronics Co., Ltd., Shenzhen, China).

### Lung Histopathology

Lung tissues were fixed in 4% formaldehyde for more than 24 h and made into a paraffin section. HE, PAS, and MT staining was performed on lung sections to assess the degree of inflammation, mucus secretion, and collagen deposition in the lungs. Histopathologic results were interpreted using scoring methods described in previous studies *via* ImageJ software (96).

### MeRIP-Seq and RNA-Seq

MeRIP-seq and RNA-seq were performed by LC-Bio Technology Co., Ltd. (Hangzhou, China). Detailed experimental procedures were described in published literature (49). In brief, three biological replicates were used in each group. Total RNA from lung tissues was isolated using Trizol (Invitrogen, Carlsbad, CA, USA) in accordance with the protocol provided by manufacturer. We then used Nanodrop ND-1000 (NanoDrop, Wilmington, DE, USA) to determine the purity and concentration of total RNA and used Bioanalyzer 2100 (Agilent, Santa Clara, CA, USA) for agarose electrophoresis to detect and verify the integrity of total RNA (RIN values >7.0). Ribosomal RNA was removed from total RNA using the Epicentre Ribo-Zero Gold Kit (Illumina, San Diego, CA, USA) and the remaining RNA was fragmented (86°C, 7 min) using RNA Fragmentation reagents (NEB, Ipswich, MA, USA). The fragmented RNA was incubated with m6A antibody (Synaptic Systems, Göttingen, Germany) as an immunoprecipitated RNA and was used for MeRIP-seq. The fragmented RNA without immunoprecipitation (IP) as an input RNA was used for RNA-seq. The IP and input RNAs were catalyzed by reverse transcriptase (Invitrogen, Carlsbad, CA, USA) to synthesize the first strand of complementary DNA (cDNA). Then, RNase H (NEB, Ipswich, MA, USA) was used to hydrolyze the RNA strand in the RNA–DNA hybridization molecule, and *Escherichia coli* DNA polymerase I (NEB, Ipswich, MA, USA) was used to synthesize the second strand of cDNA. Meanwhile, dUTP (Thermo Fisher, Waltham, MA, USA) was incorporated into the second strand. Subsequently, an A-base was added to the ends of each strand for ligating the cDNAs to the indexed adapters. After the second strand of the cDNAs containing dUTP was degraded using UDG enzyme (NEB, Ipswich, MA, USA), cDNA libraries with a fragment size of approximately 300 bp were constructed by PCR amplification. The libraries were paired-end sequenced (PE150 mode) using the Illumina Novaseq 6000 platform at LC-Bio Technology Co., Ltd. (Hangzhou, China).

### Data Processing and Statistical Analysis

Clean data in fastq format were obtained by removing adapter, repeated, and low-quality sequences from the raw reads of the IP and input samples using FASTP software (97). Clean data were aligned to the *Mus musculus* genome from Ensembl version 101 using HISAT2 (98) for obtaining the BAM files. For peak calling and identification of the differentially methylated peaks, the BAM files were analyzed using R package exomePeak (99). IGV software was used for the visualization of peaks (100) and R package ChIPseeker was used for the annotation of peaks (101). Homer software was used to search for motifs with a high confidence level in the peak region of each group of samples (http://homer.ucsd.edu/homer/motif). Mapped reads were assembled and the gene expression levels (in fragments per kilobase of transcript per million mapped reads) were assessed by StringTie (102). The differential expressions of the genes between the two groups were analyzed using the R package edgeR (103). In our study, the screening criteria for differentially methylated peaks and differentially expressed genes were |log_2_(Fold change)| > 1 and *p* < 0.05. We performed GO function and KEGG pathway enrichment analyses using the OmicStudio tools (https://www.omicstudio.cn/tool). The R package RIdeogram (36) was used to visualize the distribution of the differentially methylated m6A peaks within mRNAs. Correlation was analyzed using Pearson’s correlation. Differences between the OVA and PBS groups were analyzed using unpaired and two-tailed Student’s *t*-test, and lung function data were analyzed using two-way mixed-design ANOVA (GraphPad Prism 8.0). The data are presented as the means ± SD and differences were considered statistically significant when *p* < 0.05.

## Data Availability Statement

The data discussed in this publication have been deposited in NCBI’s Gene Expression Omnibus and are accessible through GEO Series accession number GSE179874 (https://www.ncbi.nlm.nih.gov/geo/query/acc.cgi?acc=GSE179874).

## Ethics Statement

The animal study was reviewed and approved by the Experimental Animal Ethics Committee of Fudan University School of Pharmacy (approval no. 2020-12-HSYY-WY-01).

## Author Contributions

JD and YW led and supervised the project and were involved in all aspects of the study. YW and FT conceived and designed the experiments. FT, WT, TW, JQ, YZ, XH, SW, XZ, ZT, and LY performed the experiments. FT and WT analyzed the data, interpreted the results, and prepared the figures. FT wrote the paper with input from YW. All authors commented and made edits to the manuscript. All authors contributed to the article and approved the final version.

## Funding

This work was supported by the Shanghai Science and Technology Commission Project (21S21902500), the National Natural Science Foundation of China (81774074, 82174495, 82174170), the Three-Year Action Plan (2018–2020) of Shanghai Municipality for accelerating the development of Traditional Chinese Medicine [ZY (2018–2020)-FWTX-4016], and Dong Jingcheng Expert Workstation of Yunnan Province.

## Conflict of Interest

The authors declare that the research was conducted in the absence of any commercial or financial relationships that could be construed as a potential conflict of interest.

## Publisher’s Note

All claims expressed in this article are solely those of the authors and do not necessarily represent those of their affiliated organizations, or those of the publisher, the editors and the reviewers. Any product that may be evaluated in this article, or claim that may be made by its manufacturer, is not guaranteed or endorsed by the publisher.

## References

[1] Desrosiers R Friderici K Rottman F. Identification of Methylated Nucleosides in Messenger RNA From Novikoff Hepatoma Cells. Proc Natl Acad Sci USA (1974) 71(10):3971–5. doi: 10.1073/pnas.71.10.3971 PMC434308 4372599

[2] Meyer KD Jaffrey SR. The Dynamic Epitranscriptome: N6-Methyladenosine and Gene Expression Control. Nat Rev Mol Cell Biol (2014) 15(5):313–26. doi: 10.1038/nrm3785 PMC4393108 24713629

[3] Wang X Lu Z Gomez A Hon GC Yue Y Han D. N6-Methyladenosine-Dependent Regulation of Messenger RNA Stability. Nature (2014) 505(7481):117–20. doi: 10.1038/nature12730 PMC3877715 24284625

[4] Wang X Zhao BS Roundtree IA Lu Z Han D Ma H. N(6)-Methyladenosine Modulates Messenger RNA Translation Efficiency. Cell (2015) 161(6):1388–99. doi: 10.1016/j.cell.2015.05.014 PMC4825696 26046440

[5] Boles NC Temple S. Epimetronomics: M6a Marks the Tempo of Corticogenesis. Neuron (2017) 96(4):718–20. doi: 10.1016/j.neuron.2017.11.002 29144970

[6] Shan K Zhou RM Xiang J Sun YN Liu C Lv MW. FTO Regulates Ocular Angiogenesis *via* M(6)A-YTHDF2-Dependent Mechanism. Exp Eye Res (2020) 197:108107. doi: 10.1016/j.exer.2020.108107 32531187

[7] Wang L Song C Wang N Li S Liu Q Sun Z. NADP Modulates RNA M(6)A Methylation and Adipogenesis *via* Enhancing FTO Activity. Nat Chem Biol (2020) 16(12):1394–402. doi: 10.1038/s41589-020-0601-2 32719557

[8] Lee H Bao S Qian Y Geula S Leslie J Zhang C. Stage-Specific Requirement for Mettl3-Dependent M(6)A mRNA Methylation During Haematopoietic Stem Cell Differentiation. Nat Cell Biol (2019) 21(6):700–09. doi: 10.1038/s41556-019-0318-1 PMC6556891 31061465

[9] Wang Y Sun J Lin Z Zhang W Wang S Wang W. M(6)A mRNA Methylation Controls Functional Maturation in Neonatal Murine Beta-Cells. Diabetes (2020) 69(8):1708–22. doi: 10.2337/db19-0906 32404350

[10] Kudou K Komatsu T Nogami J Maehara K Harada A Saeki H. The Requirement of Mettl3-Promoted MyoD mRNA Maintenance in Proliferative Myoblasts for Skeletal Muscle Differentiation. Open Biol (2017) 7(9):170119. doi: 10.1098/rsob.170119 28878038 PMC5627051

[11] Chen X Li X Guo J Zhang P Zeng W. The Roles of microRNAs in Regulation of Mammalian Spermatogenesis. J Anim Sci Biotechnol (2017) 8:35. doi: 10.1186/s40104-017-0166-4 28469844 PMC5410700

[12] Yang DD Chen ZH Yu K Lu JH Wu QN Wang Y. METTL3 Promotes the Progression of Gastric Cancer *via* Targeting the MYC Pathway. Front Oncol (2020) 10:115. doi: 10.3389/fonc.2020.00115 32175271 PMC7054453

[13] Liu X Gonzalez G Dai X Miao W Yuan J Huang M. Adenylate Kinase 4 Modulates the Resistance of Breast Cancer Cells to Tamoxifen Through an M(6)A-Based Epitranscriptomic Mechanism. Mol Ther (2020) 28(12):2593–604. doi: 10.1016/j.ymthe.2020.09.007 PMC7704734 32956623

[14] Liu S Li Q Li G Zhang Q Zhuo L Han X. The Mechanism of M(6)A Methyltransferase METTL3-Mediated Autophagy in Reversing Gefitinib Resistance in NSCLC Cells by Beta-Elemene. Cell Death Dis (2020) 11(11):969. doi: 10.1038/s41419-020-03148-8 33177491 PMC7658972

[15] Wang K Jiang L Zhang Y Chen C. Progression of Thyroid Carcinoma Is Promoted by the M6a Methyltransferase METTL3 Through Regulating M(6)A Methylation on TCF1. Onco Targets Ther (2020) 13:1605–12. doi: 10.2147/OTT.S234751 PMC7044742 32158230

[16] Cheng M Sheng L Gao Q Xiong Q Zhang H Wu M. The M(6)A Methyltransferase METTL3 Promotes Bladder Cancer Progression *via* AFF4/NF-Kappab/MYC Signaling Network. Oncogene (2019) 38(19):3667–80. doi: 10.1038/s41388-019-0683-z 30659266

[17] Berulava T Buchholz E Elerdashvili V Pena T Islam MR Lbik D. Changes in M6a RNA Methylation Contribute to Heart Failure Progression by Modulating Translation. Eur J Heart Fail (2020) 22(1):54–66. doi: 10.1002/ejhf.1672 31849158

[18] De Jesus DF Zhang Z Kahraman S Brown NK Chen M Hu J. M(6)A mRNA Methylation Regulates Human Beta-Cell Biology in Physiological States and in Type 2 Diabetes. Nat Metab (2019) 1(8):765–74. doi: 10.1038/s42255-019-0089-9 PMC6924515 31867565

[19] Huang R Zhang Y Bai Y Han B Ju M Chen B. N(6)-Methyladenosine Modification of Fatty Acid Amide Hydrolase Messenger RNA in Circular RNA STAG1-Regulated Astrocyte Dysfunction and Depressive-Like Behaviors. Biol Psychiatry (2020) 88(5):392–404. doi: 10.1016/j.biopsych.2020.02.018 32387133

[20] Xu Y Yuan XD Wu JJ Chen RY Xia L Zhang M. The N6-Methyladenosine mRNA Methylase METTL14 Promotes Renal Ischemic Reperfusion Injury *via* Suppressing YAP1. J Cell Biochem (2020) 121(1):524–33. doi: 10.1002/jcb.29258 31318098

[21] Shulman Z Stern-Ginossar N. The RNA Modification N(6)-Methyladenosine as a Novel Regulator of the Immune System. Nat Immunol (2020) 21(5):501–12. doi: 10.1038/s41590-020-0650-4 32284591

[22] Liu Y Liu Z Tang H Shen Y Gong Z Xie N. The N(6)-Methyladenosine (M(6)A)-Forming Enzyme METTL3 Facilitates M1 Macrophage Polarization Through the Methylation of STAT1 mRNA. Am J Physiol Cell Physiol (2019) 317(4):C762–75. doi: 10.1152/ajpcell.00212.2019 31365297

[23] Li HB Tong J Zhu S Batista PJ Duffy EE Zhao J. M(6)A mRNA Methylation Controls T Cell Homeostasis by Targeting the IL-7/STAT5/SOCS Pathways. Nature (2017) 548(7667):338–42. doi: 10.1038/nature23450 PMC5729908 28792938

[24] Lu TX Zheng Z Zhang L Sun HL Bissonnette M Huang H. A New Model of Spontaneous Colitis in Mice Induced by Deletion of an RNA M(6)A Methyltransferase Component METTL14 in T Cells. Cell Mol Gastroenterol Hepatol (2020) 10(4):747–61. doi: 10.1016/j.jcmgh.2020.07.001 PMC7498954 32634481

[25] Yu JT Hu XW Chen HY Yang Q Li HD Dong YH. DNA Methylation of FTO Promotes Renal Inflammation by Enhancing M(6)A of PPAR-Alpha in Alcohol-Induced Kidney Injury. Pharmacol Res (2021) 163:105286. doi: 10.1016/j.phrs.2020.105286 33157234

[26] Guo M Yan R Ji Q Yao H Sun M Duan L. IFN Regulatory Factor-1 Induced Macrophage Pyroptosis by Modulating M6a Modification of Circ_0029589 in Patients With Acute Coronary Syndrome. Int Immunopharmacol (2020) 86:106800. doi: 10.1016/j.intimp.2020.106800 32674051

[27] Lambrecht BN Hammad H Fahy JV. The Cytokines of Asthma. Immunity (2019) 50(4):975–91. doi: 10.1016/j.immuni.2019.03.018 30995510

[28] Kuruvilla ME Lee FE Lee GB. Understanding Asthma Phenotypes, Endotypes, and Mechanisms of Disease. Clin Rev Allergy Immunol (2019) 56(2):219–33. doi: 10.1007/s12016-018-8712-1 PMC6411459 30206782

[29] Ladjemi MZ Gras D Dupasquier S Detry B Lecocq M Garulli C. Bronchial Epithelial IgA Secretion Is Impaired in Asthma. Role of IL-4/IL-13. Am J Respir Crit Care Med (2018) 197(11):1396–409. doi: 10.1164/rccm.201703-0561OC 29652177

[30] Nials AT Uddin S. Mouse Models of Allergic Asthma: Acute and Chronic Allergen Challenge. Dis Model Mech (2008) 1(4-5):213–20. doi: 10.1242/dmm.000323 PMC2590830 19093027

[31] Blanchet MR Gold MJ McNagny KM. Mouse Models to Evaluate the Function of Genes Associated With Allergic Airway Disease. Curr Opin Allergy Clin Immunol (2012) 12(5):467–74. doi: 10.1097/ACI.0b013e328357cc17 22885889

[32] Kaczmarek KA Clifford RL Knox AJ. Epigenetic Changes in Airway Smooth Muscle as a Driver of Airway Inflammation and Remodeling in Asthma. Chest (2019) 155(4):816–24. doi: 10.1016/j.chest.2018.10.038 30414795

[33] Durham AL Wiegman C Adcock IM. Epigenetics of Asthma. Biochim Biophys Acta (2011) 1810(11):1103–9. doi: 10.1016/j.bbagen.2011.03.006 21397662

[34] Qi C Xu CJ Koppelman GH. The Role of Epigenetics in the Development of Childhood Asthma. Expert Rev Clin Immunol (2019) 15(12):1287–302. doi: 10.1080/1744666X.2020.1686977 31674254

[35] Zhao BS Roundtree IA He C. Post-Transcriptional Gene Regulation by mRNA Modifications. Nat Rev Mol Cell Biol (2017) 18(1):31–42. doi: 10.1038/nrm.2016.132 27808276 PMC5167638

[36] Hao Z Lv D Ge Y Shi J Weijers D Yu G. RIdeogram: Drawing SVG Graphics to Visualize and Map Genome-Wide Data on the Idiograms. PeerJ Comput Sci (2020) 6:e251. doi: 10.7717/peerj-cs.251 PMC7924719 33816903

[37] Xuan JJ Sun WJ Lin PH Zhou KR Liu S Zheng LL. RMBase V2.0: Deciphering the Map of RNA Modifications From Epitranscriptome Sequencing Data. Nucleic Acids Res (2018) 46(D1):D327–34. doi: 10.1093/nar/gkx934 PMC5753293 29040692

[38] Fu Y Dominissini D Rechavi G He C. Gene Expression Regulation Mediated Through Reversible M(6)A RNA Methylation. Nat Rev Genet (2014) 15(5):293–306. doi: 10.1038/nrg3724 24662220

[39] Dai B Sun F Cai X Li C Liu H Shang Y. Significance of RNA N6-Methyladenosine Regulators in the Diagnosis and Subtype Classification of Childhood Asthma Using the Gene Expression Omnibus Database. Front Genet (2021) 12:634162. doi: 10.3389/fgene.2021.634162 33763115 PMC7982807

[40] Kim H Lee YS Kim SM Jang S Choi H Lee JW. RNA Demethylation by FTO Stabilizes the FOXJ1 mRNA for Proper Motile Ciliogenesis. Dev Cell (2021) 56(8):1118–30.e6. doi: 10.1016/j.devcel.2021.03.006 33761320

[41] Yuan Q Zhu H Liu H Wang M Chu H Zhang Z. METTL3 Regulates PM2.5-Induced Cell Injury by Targeting OSGIN1 in Human Airway Epithelial Cells. J Hazard Mater (2021) 415:125573. doi: 10.1016/j.jhazmat.2021.125573 33730643

[42] Koya T Kodama T Takeda K Miyahara N Yang ES Taube C. Importance of Myeloid Dendritic Cells in Persistent Airway Disease After Repeated Allergen Exposure. Am J Respir Crit Care Med (2006) 173(1):42–55. doi: 10.1164/rccm.200505-783OC 16192450 PMC2662981

[43] Chupp GL Kaur R Mainardi A. New Therapies for Emerging Endotypes of Asthma. Annu Rev Med (2020) 71:289–302. doi: 10.1146/annurev-med-041818-020630 31689153

[44] Wuniqiemu T Qin J Teng F Nabijan M Cui J Yi L. Quantitative Proteomic Profiling of Targeted Proteins Associated With Loki Zupa Decoction Treatment in OVA-Induced Asthmatic Mice. J Ethnopharmacol (2021) 266:113343. doi: 10.1016/j.jep.2020.113343 32991972

[45] Tang W Dong M Teng F Cui J Zhu X Wang W. TMT-Based Quantitative Proteomics Reveals Suppression of SLC3A2 and ATP1A3 Expression Contributes to the Inhibitory Role of Acupuncture on Airway Inflammation in an OVA-Induced Mouse Asthma Model. BioMed Pharmacother (2021) 134:111001. doi: 10.1016/j.biopha.2020.111001 33341053

[46] Luo Z Zhang Z Tai L Zhang L Sun Z Zhou L. Comprehensive Analysis of Differences of N(6)-Methyladenosine RNA Methylomes Between High-Fat-Fed and Normal Mouse Livers. Epigenomics (2019) 11(11):1267–82. doi: 10.2217/epi-2019-0009 31290331

[47] Lu Z Liu J Yuan C Jin M Quan K Chu M. M(6)A mRNA Methylation Analysis Provides Novel Insights Into Heat Stress Responses in the Liver Tissue of Sheep. Genomics (2021) 113(1 Pt 2):484–92. doi: 10.1016/j.ygeno.2020.09.038 32976974

[48] Yang C Zhao K Zhang J Wu X Sun W Kong X. Comprehensive Analysis of the Transcriptome-Wide M6a Methylome of Heart *via* MeRIP After Birth: Day 0 *vs.* Day 7. Front Cardiovasc Med (2021) 8:633631. doi: 10.3389/fcvm.2021.633631 33829047 PMC8019948

[49] Han Z Yang B Wang Q Hu Y Wu Y Tian Z. Comprehensive Analysis of the Transcriptome-Wide M(6)A Methylome in Invasive Malignant Pleomorphic Adenoma. Cancer Cell Int (2021) 21(1):142. doi: 10.1186/s12935-021-01839-6 33653351 PMC7923655

[50] Zhang Z Wang Q Zhang M Zhang W Zhao L Yang C. Comprehensive Analysis of the Transcriptome-Wide M6a Methylome in Colorectal Cancer by MeRIP Sequencing. Epigenetics (2021) 16(4):425–35. doi: 10.1080/15592294.2020.1805684 PMC7993153 32749190

[51] Choy DF Modrek B Abbas AR Kummerfeld S Clark HF Wu LC. Gene Expression Patterns of Th2 Inflammation and Intercellular Communication in Asthmatic Airways. J Immunol (2011) 186(3):1861–9. doi: 10.4049/jimmunol.1002568 PMC3981556 21187436

[52] Tebroke J Lieverse JE Safholm J Schulte G Nilsson G Ronnberg E. Wnt-3a Induces Cytokine Release in Human Mast Cells. Cells (2019) 8(11):1372. doi: 10.3390/cells8111372 PMC6912728 31683769

[53] Reuter S Beckert H Taube C. Take the Wnt Out of the Inflammatory Sails: Modulatory Effects of Wnt in Airway Diseases. Lab Invest (2016) 96(2):177–85. doi: 10.1038/labinvest.2015.143 26595171

[54] Reuter S Martin H Beckert H Bros M Montermann E Belz C. The Wnt/beta-Catenin Pathway Attenuates Experimental Allergic Airway Disease. J Immunol (2014) 193(2):485–95. doi: 10.4049/jimmunol.1400013 24929002

[55] Huang TJ MacAry PA Eynott P Moussavi A Daniel KC Askenase PW. Allergen-Specific Th1 Cells Counteract Efferent Th2 Cell-Dependent Bronchial Hyperresponsiveness and Eosinophilic Inflammation Partly *via* IFN-Gamma. J Immunol (2001) 166(1):207–17. doi: 10.4049/jimmunol.166.1.207 11123294

[56] Qiu YY Zhang YW Qian XF Bian T. miR-371, miR-138, miR-544, miR-145, and miR-214 Could Modulate Th1/Th2 Balance in Asthma Through the Combinatorial Regulation of Runx3. Am J Transl Res (2017) 9(7):3184–99. PMC5553871 28804539

[57] Takaoka A Tanaka Y Tsuji T Jinushi T Hoshino A Asakura Y. A Critical Role for Mouse CXC Chemokine(s) in Pulmonary Neutrophilia During Th Type 1-Dependent Airway Inflammation. J Immunol (2001) 167(4):2349–53. doi: 10.4049/jimmunol.167.4.2349 11490024

[58] Sugimoto T Ishikawa Y Yoshimoto T Hayashi N Fujimoto J Nakanishi K. Interleukin 18 Acts on Memory T Helper Cells Type 1 to Induce Airway Inflammation and Hyperresponsiveness in a Naive Host Mouse. J Exp Med (2004) 199(4):535–45. doi: 10.1084/jem.20031368 PMC2211833 14970180

[59] Shinjo Y Makide K Satoh K Fukami F Inoue A Kano K. Lysophosphatidylserine Suppresses IL-2 Production in CD4 T Cells Through LPS3/GPR174. Biochem Biophys Res Commun (2017) 494(1-2):332–38. doi: 10.1016/j.bbrc.2017.10.028 29017923

[60] Barnes MJ Li CM Xu Y An J Huang Y Cyster JG. The Lysophosphatidylserine Receptor GPR174 Constrains Regulatory T Cell Development and Function. J Exp Med (2015) 212(7):1011–20. doi: 10.1084/jem.20141827 PMC4493414 26077720

[61] Recio C Lucy D Purvis GSD Iveson P Zeboudj L Iqbal AJ. Activation of the Immune-Metabolic Receptor GPR84 Enhances Inflammation and Phagocytosis in Macrophages. Front Immunol (2018) 9:1419. doi: 10.3389/fimmu.2018.01419 29973940 PMC6019444

[62] Wang J Wu X Simonavicius N Tian H Ling L. Medium-Chain Fatty Acids as Ligands for Orphan G Protein-Coupled Receptor GPR84. J Biol Chem (2006) 281(45):34457–64. doi: 10.1074/jbc.M608019200 16966319

[63] Tsunemi Y Saeki H Nakamura K Sekiya T Hirai K Fujita H. Interleukin-12 P40 Gene (IL12B) 3'-Untranslated Region Polymorphism is Associated With Susceptibility to Atopic Dermatitis and Psoriasis Vulgaris. J Dermatol Sci (2002) 30(2):161–6. doi: 10.1016/s0923-1811(02)00072-5 12413772

[64] Osborn O Oh DY McNelis J Sanchez-Alavez M Talukdar S Lu M. G Protein-Coupled Receptor 21 Deletion Improves Insulin Sensitivity in Diet-Induced Obese Mice. J Clin Invest (2012) 122(7):2444–53. doi: 10.1172/JCI61953 PMC3386820 22653059

[65] Uza N Nakase H Yamamoto S Yoshino T Takeda Y Ueno S. SR-PSOX/CXCL16 Plays a Critical Role in the Progression of Colonic Inflammation. Gut (2011) 60(11):1494–505. doi: 10.1136/gut.2010.221879 21471570

[66] Lai AY Kondo M. T and B Lymphocyte Differentiation From Hematopoietic Stem Cell. Semin Immunol (2008) 20(4):207–12. doi: 10.1016/j.smim.2008.05.002 PMC2570370 18583148

[67] Weinstein JA Zeng X Chien YH Quake SR. Correlation of Gene Expression and Genome Mutation in Single B-Cells. PloS One (2013) 8(6):e67624. doi: 10.1371/journal.pone.0067624 23840752 PMC3695916

[68] McElrath TF Cantonwine DE Gray KJ Mirzakhani H Doss RC Khaja N. Late First Trimester Circulating Microparticle Proteins Predict the Risk of Preeclampsia < 35 Weeks and Suggest Phenotypic Differences Among Affected Cases. Sci Rep (2020) 10(1):17353. doi: 10.1038/s41598-020-74078-w 33087742 PMC7578826

[69] Achkar JM Cortes L Croteau P Yanofsky C Mentinova M Rajotte I. Host Protein Biomarkers Identify Active Tuberculosis in HIV Uninfected and Co-Infected Individuals. EBioMedicine (2015) 2(9):1160–8. doi: 10.1016/j.ebiom.2015.07.039 PMC4588417 26501113

[70] McNicol A Israels SJ. Beyond Hemostasis: The Role of Platelets in Inflammation, Malignancy and Infection. Cardiovasc Hematol Disord Drug Targets (2008) 8(2):99–117. doi: 10.2174/187152908784533739 18537597

[71] Kelly EA Koziol-White CJ Clay KJ Liu LY Bates ME Bertics PJ. Potential Contribution of IL-7 to Allergen-Induced Eosinophilic Airway Inflammation in Asthma. J Immunol (2009) 182(3):1404–10. doi: 10.4049/jimmunol.182.3.1404 PMC2851244 19155487

[72] Zhang X Huo L Song L Hu Z Wang X Han Y. Dominant Negative FADD/MORT1 Inhibits the Development of Intestinal Intraepithelial Lymphocytes With a Marked Defect on CD8alphaalpha+TCRgammadelta+ T Cells. Front Immunol (2018) 9:2038. doi: 10.3389/fimmu.2018.02038 30250469 PMC6139313

[73] Tarakhovsky A Kanner SB Hombach J Ledbetter JA Muller W Killeen N. A Role for CD5 in TCR-Mediated Signal Transduction and Thymocyte Selection. Science (1995) 269(5223):535–7. doi: 10.1126/science.7542801 7542801

[74] Glisovic T Bachorik JL Yong J Dreyfuss G. RNA-Binding Proteins and Post-Transcriptional Gene Regulation. FEBS Lett (2008) 582(14):1977–86. doi: 10.1016/j.febslet.2008.03.004 PMC2858862 18342629

[75] Zhang Z Luo K Zou Z Qiu M Tian J Sieh L. Genetic Analyses Support the Contribution of mRNA N(6)-Methyladenosine (M(6)A) Modification to Human Disease Heritability. Nat Genet (2020) 52(9):939–49. doi: 10.1038/s41588-020-0644-z PMC7483307 32601472

[76] Yamazumi Y Sasaki O Suyama-Fuchino S Kohu K Kamoshida Y Harada H. The RNA-Binding Protein Mex-3B Plays Critical Roles in the Development of Steroid-Resistant Neutrophilic Airway Inflammation. Biochem Biophys Res Commun (2019) 519(2):220–26. doi: 10.1016/j.bbrc.2019.08.158 31493864

[77] Kennedy JM Fodil N Torre S Bongfen SE Olivier JF Leung V. CCDC88B Is a Novel Regulator of Maturation and Effector Functions of T Cells During Pathological Inflammation. J Exp Med (2014) 211(13):2519–35. doi: 10.1084/jem.20140455 PMC4267237 25403443

[78] Fodil N Moradin N Leung V Olivier JF Radovanovic I Jeyakumar T. CCDC88B is Required for Pathogenesis of Inflammatory Bowel Disease. Nat Commun (2017) 8(1):932. doi: 10.1038/s41467-017-01381-y 29030607 PMC5640600

[79] Yoo HJ Hwang WC Min DS. Targeting of Phospholipase D1 Ameliorates Collagen-Induced Arthritis *via* Modulation of Treg and Th17 Cell Imbalance and Suppression of Osteoclastogenesis. Int J Mol Sci (2020) 21(9):3230. doi: 10.3390/ijms21093230 PMC7247592 32370217

[80] Gavin AL Huang D Huber C Martensson A Tardif V Skog PD. PLD3 and PLD4 are Single-Stranded Acid Exonucleases That Regulate Endosomal Nucleic-Acid Sensing. Nat Immunol (2018) 19(9):942–53. doi: 10.1038/s41590-018-0179-y PMC6105523 30111894

[81] Akizuki S Ishigaki K Kochi Y Law SM Matsuo K Ohmura K. PLD4 is a Genetic Determinant to Systemic Lupus Erythematosus and Involved in Murine Autoimmune Phenotypes. Ann Rheum Dis (2019) 78(4):509–18. doi: 10.1136/annrheumdis-2018-214116 30679154

[82] Lin J Kumari S Kim C Van TM Wachsmuth L Polykratis A. RIPK1 Counteracts ZBP1-Mediated Necroptosis to Inhibit Inflammation. Nature (2016) 540(7631):124–28. doi: 10.1038/nature20558 PMC5755685 27819681

[83] Kuriakose T Kanneganti TD. ZBP1: Innate Sensor Regulating Cell Death and Inflammation. Trends Immunol (2018) 39(2):123–34. doi: 10.1016/j.it.2017.11.002 PMC5863909 29236673

[84] Maelfait J Liverpool L Rehwinkel J. Nucleic Acid Sensors and Programmed Cell Death. J Mol Biol (2020) 432(2):552–68. doi: 10.1016/j.jmb.2019.11.016 PMC7322524 31786265

[85] Devos M Tanghe G Gilbert B Dierick E Verheirstraeten M Nemegeer J. Sensing of Endogenous Nucleic Acids by ZBP1 Induces Keratinocyte Necroptosis and Skin Inflammation. J Exp Med (2020) 217(7):e20191913. doi: 10.1084/jem.20191913 32315377 PMC7336309

[86] Gessier F Preuss I Yin H Rosenkilde MM Laurent S Endres R. Identification and Characterization of Small Molecule Modulators of the Epstein-Barr Virus-Induced Gene 2 (EBI2) Receptor. J Med Chem (2014) 57(8):3358–68. doi: 10.1021/jm4019355 24678947

[87] Li J Lu E Yi T Cyster JG. EBI2 Augments Tfh Cell Fate by Promoting Interaction With IL-2-Quenching Dendritic Cells. Nature (2016) 533(7601):110–4. doi: 10.1038/nature17947 PMC4883664 27147029

[88] Hannedouche S Zhang J Yi T Shen W Nguyen D Pereira JP. Oxysterols Direct Immune Cell Migration *via* EBI2. Nature (2011) 475(7357):524–7. doi: 10.1038/nature10280 PMC4297623 21796212

[89] Liu C Yang XV Wu J Kuei C Mani NS Zhang L. Oxysterols Direct B-Cell Migration Through EBI2. Nature (2011) 475(7357):519–23. doi: 10.1038/nature10226 21796211

[90] Gu L Tseng S Horner RM Tam C Loda M Rollins BJ. Control of TH2 Polarization by the Chemokine Monocyte Chemoattractant Protein-1. Nature (2000) 404(6776):407–11. doi: 10.1038/35006097 10746730

[91] David BA Kubes P. Exploring the Complex Role of Chemokines and Chemoattractants *In Vivo* on Leukocyte Dynamics. Immunol Rev (2019) 289(1):9–30. doi: 10.1111/imr.12757 30977202

[92] Tsou CL Peters W Si Y Slaymaker S Aslanian AM Weisberg SP. Critical Roles for CCR2 and MCP-3 in Monocyte Mobilization From Bone Marrow and Recruitment to Inflammatory Sites. J Clin Invest (2007) 117(4):902–9. doi: 10.1172/JCI29919 PMC1810572 17364026

[93] Provoost S Maes T Joos GF Tournoy KG. Monocyte-Derived Dendritic Cell Recruitment and Allergic T(H)2 Responses After Exposure to Diesel Particles are CCR2 Dependent. J Allergy Clin Immunol (2012) 129(2):483–91. doi: 10.1016/j.jaci.2011.07.051 21906792

[94] Sokol CL Camire RB Jones MC Luster AD. The Chemokine Receptor CCR8 Promotes the Migration of Dendritic Cells Into the Lymph Node Parenchyma to Initiate the Allergic Immune Response. Immunity (2018) 49(3):449–63 e6. doi: 10.1016/j.immuni.2018.07.012 30170811 PMC6192021

[95] Haspeslagh E Debeuf N Hammad H Lambrecht BN. Murine Models of Allergic Asthma. Methods Mol Biol (2017) 1559:121–36. doi: 10.1007/978-1-4939-6786-5_10 28063042

[96] Ma Y Ge A Zhu W Liu YN Ji NF Zha WJ. Morin Attenuates Ovalbumin-Induced Airway Inflammation by Modulating Oxidative Stress-Responsive MAPK Signaling. Oxid Med Cell Longev (2016) 2016:5843672. doi: 10.1155/2016/5843672 26783416 PMC4691473

[97] Chen S Zhou Y Chen Y Gu J. Fastp: An Ultra-Fast All-in-One FASTQ Preprocessor. Bioinformatics (2018) 34(17):i884–90. doi: 10.1093/bioinformatics/bty560 PMC6129281 30423086

[98] Kim D Langmead B Salzberg SL. HISAT: A Fast Spliced Aligner With Low Memory Requirements. Nat Methods (2015) 12(4):357–60. doi: 10.1038/nmeth.3317 PMC4655817 25751142

[99] Meng J Lu Z Liu H Zhang L Zhang S Chen Y. A Protocol for RNA Methylation Differential Analysis With MeRIP-Seq Data and Exomepeak R/Bioconductor Package. Methods (2014) 69(3):274–81. doi: 10.1016/j.ymeth.2014.06.008 PMC4194139 24979058

[100] Robinson JT Thorvaldsdottir H Winckler W Guttman M Lander ES Getz G. Integrative Genomics Viewer. Nat Biotechnol (2011) 29(1):24–6. doi: 10.1038/nbt.1754 PMC3346182 21221095

[101] Yu G Wang LG He QY. ChIPseeker: An R/Bioconductor Package for ChIP Peak Annotation, Comparison and Visualization. Bioinformatics (2015) 31(14):2382–3. doi: 10.1093/bioinformatics/btv145 25765347

[102] Pertea M Pertea GM Antonescu CM Chang TC Mendell JT Salzberg SL. StringTie Enables Improved Reconstruction of a Transcriptome From RNA-Seq Reads. Nat Biotechnol (2015) 33(3):290–5. doi: 10.1038/nbt.3122 PMC4643835 25690850

[103] Robinson MD McCarthy DJ Smyth GK. Edger: A Bioconductor Package for Differential Expression Analysis of Digital Gene Expression Data. Bioinformatics (2010) 26(1):139–40. doi: 10.1093/bioinformatics/btp616 PMC2796818 19910308

